# A Highly Conductive Halospinel Cathode for All-Solid-State Batteries

**DOI:** 10.1021/acsenergylett.5c02476

**Published:** 2025-10-31

**Authors:** Julian F. Baumgärtner, Daniel Isler, Hung Quoc Nguyen, Matthias Klimpel, Jaka Šivavec, Chris Černe, Dmitry Chernyshov, Wouter van Beek, Daniel Rettenwander, Kostiantyn V. Kravchyk, Maksym V. Kovalenko

**Affiliations:** † Laboratory of Inorganic Chemistry, Department of Chemistry and Applied Biosciences, 27219 ETH Zürich, CH-8093 Zürich, Switzerland; ‡ Laboratory for Thin Films and Photovoltaics, Empa - Swiss Federal Laboratories for Materials Science and Technology, CH-8600 Dübendorf, Switzerland; § Department of Material Science and Engineering, 8018 NTNU Norwegian University of Science and Technology, 7034 Trondheim, Norway; ∥ Swiss−Norwegian Beam Lines at the European Synchrotron Radiation Facility, 38000 Grenoble, France; ⊥ Christian Doppler Laboratory for Solid-State Batteries, NTNU Norwegian University of Science and Technology, 7034 Trondheim, Norway; # Austrian Institute of Technology GmbH, Center for Transport Technologies, Battery Technologies, Vienna 1210, Austria

## Abstract

High-power lithium-ion batteries (LIBs) rely on highly ionically and electronically conductive cathode active materials (CAMs). While oxospinels meet these criteria and are therefore widely employed in state-of-the-art LIBs, we demonstrate that halospinels offer greatly enhanced transport properties and enable the incorporation of earth-abundant transition metals, such as iron. Using spinel-type Li_2–*x*_FeCl_4_ (0 < *x* ≤ 1, LFC) as a model CAM in an all-solid-state battery (ASSBs), we show that its intrinsically high ionic-electronic conductivity enables the fabrication of cathodes composed of micron-sized CAM particles with high areal capacity (>2 mA h cm^–2^) at practical current densities (0.5 mA cm^–2^) over 200 cycles. Our findings position LFC as a promising CAM, paving the way for cost-effective, high-performance ASSBs.

## Graphical abstract

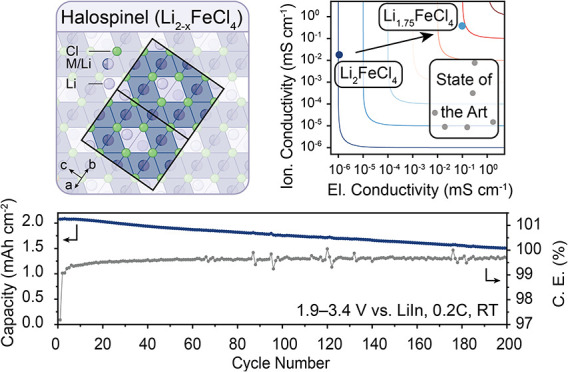

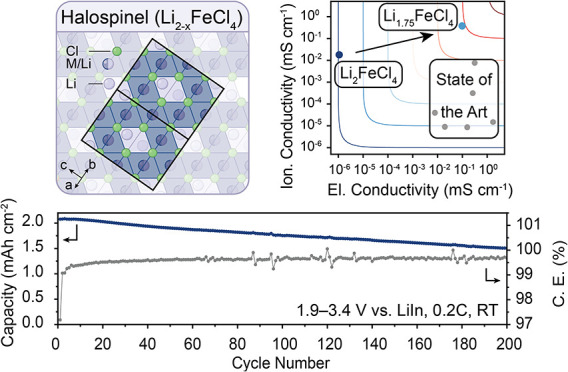

To meet the growing demands of modern energy storage, cathode active materials (CAMs) in Li-ion batteries (LIBs) not only need to offer high energy density, but also enable fast charge and discharge.^1–3^ To this end, the CAM must facilitate both rapid electronic and ionic transport at the lattice scale, since chemical diffusion will be rate-limited by the slower species.^4,5^ Efficient charge transport relies on both high charge carrier mobility and concentration, which has led to well-established design principles for CAMs: First, high Li-ion and electron mobility necessitate crystal structures with two percolating networks – one for Li-ions and one for electrons. Li-ion mobility is further enhanced by a shallow free-energy landscape along the migration pathway,^6,7^ while electronic mobility in late 3d transition metal (TM) salts is facilitated via small polaron hopping,^8–10^ which requires close proximity between adjacent metal centers,^11^ as provided in edge-sharing TMX_6_ octahedra (where X is an anionic ligand) (Figure 1A,B). Second, charge carrier concentrations can be increased by introducing vacancies into the Li-ion conducting networks, while the presence of TMs in multiple oxidation states similarly improves the electronic carrier concentration.

**Figure 1:**
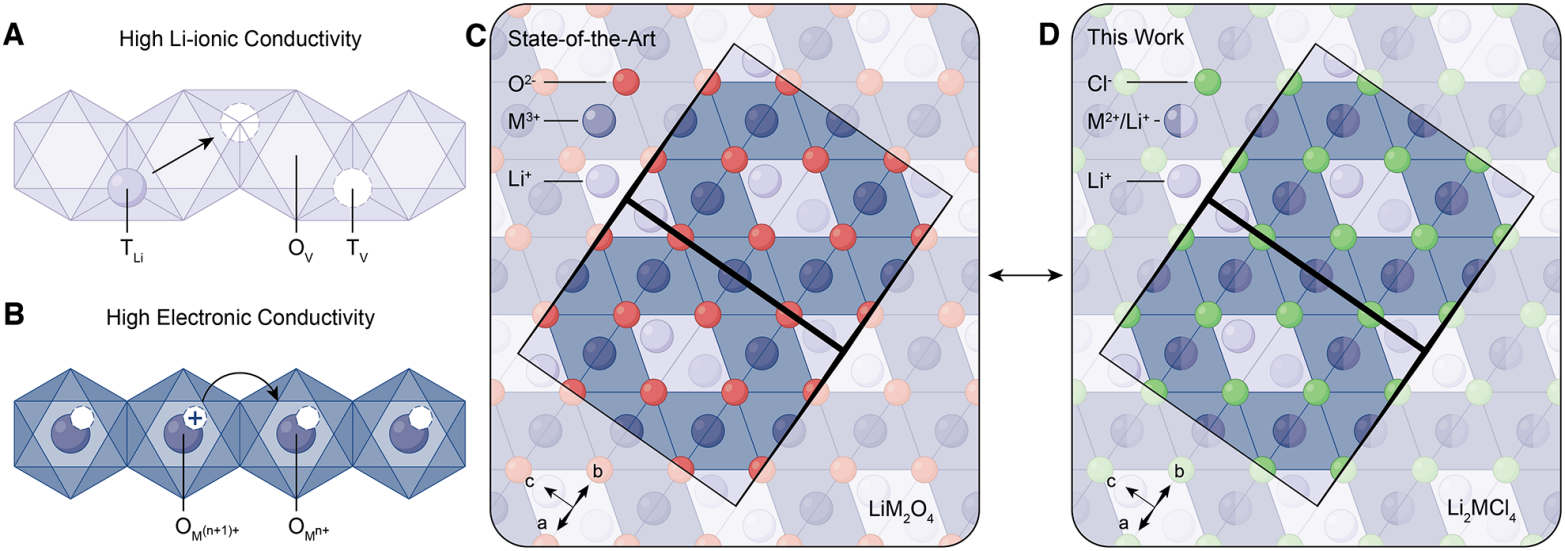
Design principles of highly conductive CAMs. (A, B) Structural features of highly conductive cathode active materials. (C, D) Schematic 2D representation of oxo- (C) and chlorospinel (D) structures with the unit cell highlighted by black borders.

Spinel (Figure 1C)^12,13^ and layered^14,15^ CAMs such as lithium transition metal oxides (Li_*x*_TMO_2_, 0 < *x* ≤ 1) have become synonymous with these design principles as they enable both ionic and electronic percolation, as well as high carrier mobility.^16^ Meanwhile, the charge carrier concentration within these structures naturally increases during (de)­lithiation due to their wide Li-ion solubility ranges.^17–19^ Partially removing Li-ions from the structure, therefore, vastly increases the number of available vacancies for Li-ion hopping, whereas the electronic conductivity may also increase depending on the d-electron configuration of the TM. Yet, the necessity for the TM to exist in the +IV oxidation state in Li_*x*_TMO_2_ with sufficiently high lithiation potentials fundamentally limits the choice of redox-active 3d TMs to Mn, Co, and Ni. While Co and Ni are scarce in the earth’s crust and therefore expensive, Mn-based CAMs, such as spinel-type Li_*x*_Mn_2_O_4_, suffer from Jahn–Teller distortions upon discharge,^20^ severely hindering their practical application. To integrate TMs with a preferred +II/+III redox couple into a spinel or layered framework, the charge of the anion sublattice must be reduced.

We address this limitation by employing halospinels (Li_2–*x*_TMCl_4_, 0 < *x* ≤ 1) with a reduced anionic charge as CAMs in all-solid-state Li-ion batteries (ASSBs), thereby extending the range of viable TMs (Figure 1D). We selected Li_2_FeCl_4_ (LFC) as a model system because of its abundant and sustainable composition, high charge-storage capacity and favorable lithiation potential.^21–26^ We further reasoned that chlorides, forming a softer anion sublattice than oxides,^27,28^ may provide even higher ionic conductivity than state-of-the-art Li_*x*_TMO_2_ CAMs. By leveraging Landau theory to analyze the order–disorder phase transition between the orthorhombic room temperature (RT) modification of Li_2_FeCl_4_ (o-LFC),^29–31^ and its high-temperature spinel polymorph (c-LFC),^32^ we depart from previous reports to demonstrate that o-LFC retains the preferrable 3D Li-ion conduction network of the spinel motif. We further map the phase diagram of Li_2–*x*_FeCl_4_ (0 ≤ *x* ≤ 1) upon delithiation and identify a partially delithiated intermediate orthorhombic spinel phase (o-Li_1.75_FeCl_4_). Compared to o-Li_2_FeCl_4_, o-Li_1.75_FeCl_4_ exhibits superior ionic conductivity (0.4 mS cm^–1^), and the electronic conductivity is boosted by over 4 orders of magnitude to 0.1 mS cm^–1^, far surpassing the ionic conductivity of state-of-the-art Li_*x*_TMO_2_ CAMs, while maintaining comparable electronic conductivity.^33–39^ Moreover, superior charge transport is preserved throughout (de)­lithiation by the continued presence of the highly conductive o-Li_1.75–*x*_FeCl_4_ phase, owing to the unique two-phase/solid-solution lithiation mechanism of LFC. These advances enable ASSBs with exceptional areal capacity (>2 mA h cm^–2^) at practical rates (0.5 mA cm^–2^) and a prolonged cycle life of 200 cycles. Our findings firmly establish LFC as a commercially viable high-performance CAM and enable the realization of cost-effective ASSBs.

## Spinel Structure of Li_2_FeCl_4_

LFC adopts a cubic inverse spinel structure at high temperatures (c-LFC, *Fd* 3 *m*), with Fe­(II)- and Li-ions statistically occupying the octahedral site, and a Li-ion occupying the tetrahedral site.^32^ c-LFC may therefore display high ionic and electronic mobility according to the design principles discussed above (Figure 1). Meanwhile, its room temperature orthorhombic modification (o-LFC) remains debated,^29–31^ hindering a rational understanding of its properties. o-LFC has been previously described with Fe­(II)- and Li-ions each occupying 1/8 of the octahedral sites to form 1D chains of edge-sharing octahedra within the f.c.c. Cl-ion sublattice (Figure S2).^29–31^ However, no consensus has been reached about the equilibrium site of the mobile Li-ion, despite its importance for the Li-ion percolating network. Previous reports suggested a Li-ion on an octahedral site (4f in *Cmmm*, Figure S1B),^29,30^ or on a split position between the octahedral and tetrahedral site (8i in *Imma*, Figure S1A),^31^ both of which would result in 1D Li-ion diffusion pathways.

As-prepared o-LFC was synthesized mechanochemically from LiCl and FeCl_2_. Rietveld refinement confirmed the presence of o-LFC alongside minor Li_6_FeCl_8_ impurities (Figure S2, Tables S1–S6). Due to the broad reflections of as-prepared o-LFC and the weak scattering intensity of Li, both models (*Cmmm* and *Imma*) show similar agreement with the experimental pattern and cannot be distinguished (Supplementary Text 1). To gain insights into the structure of o-LFC, and the equilibrium site of the mobile Li-ion within it, we studied its relationship to the high-temperature cubic polymorph (c-LFC). If the phase transition between c-LFC and o-LFC is continuous (second-order), Landau theory severely narrows down the possible structures for o-LFC based on the required group-subgroup relationships between the two phases.^40^ Crucially, the symmetry constraints would imply that the 3D percolating network of the spinel structure is retained, which is commensurate with the *Imma* space group, but only if the Li-ion remains on the tetrahedral site (4e), and not on the 8i site.^41,42^

To study the phase transition of o-LFC, *in situ* synchrotron XRD (SXRD) of as-prepared o-LFC was performed during heating (Figure S3A–F). Upon heating to 134 °C, the sample is thermally annealed, resulting in reduced reflection widths and confirming the coexistence of o-LFC and Li_6_FeCl_8_ in the as-prepared sample by two separate sets of reflections (Figure S3A–C). At the same temperature, a loss of the superstructure reflections marked the phase transition from cation-ordered o-LFC to cation-disordered c-LFC. Above 250 °C, the disappearance of Li_6_FeCl_8_, indicated the growth of stoichiometric spinel-type c-LFC, in line with the previously suggested phase diagram of LiCl-FeCl_2_.^43^

The phase transition was subsequently tracked close to thermal equilibrium at a slow cooling rate of −0.1 °C min^–1^ (Figure 2A, B). Second-order phase transitions show a continuous (and often linear in mean-field theory) increase of the superstructure reflection intensity associated with the ordered low-symmetry phase below the critical temperature *T*_c_ (Supplementary Text 2). Indeed, the 002 superstructure reflection associated with cation ordering in o-LFC reappeared continuously and linearly below 130 °C (Figure 2B), confirming the second-order phase transition between c-LFC and o-LFC. We note that before the phase transition the small reflection at almost the same position is caused by the Li_6_FeCl_8_ impurity phase.

**Figure 2:**
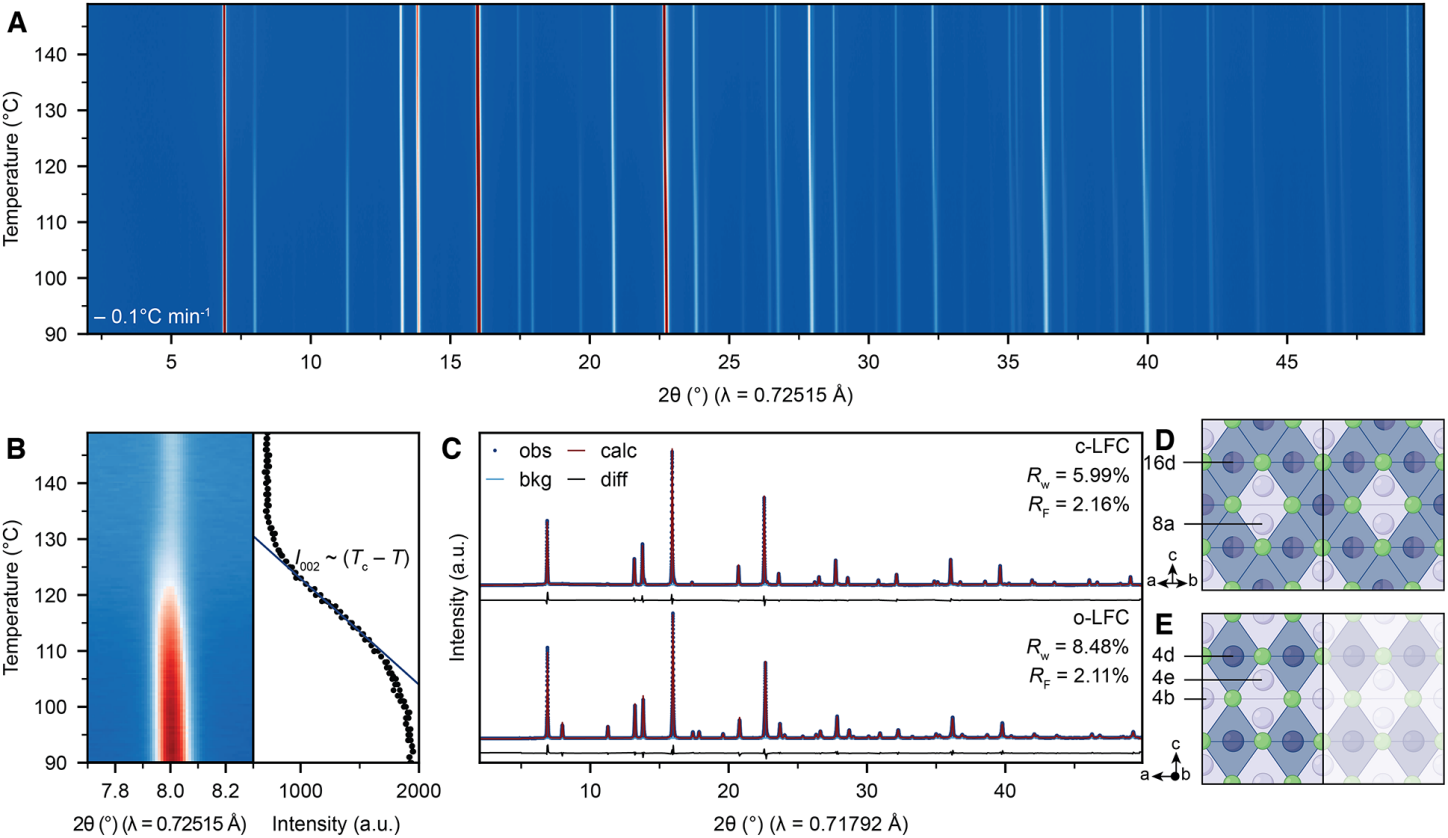
Spinel structure of Li_2_FeCl_4_. (A, B) *In-situ* SXRD of Li_2_FeCl_4_ during cooldown at −0.1 °C min^–1^ (A). The continuous linear intensity evolution of the 002 reflection confirms a 2nd order phase transition according to Landau theory (B). (C–E) SXRD refinements for spinel-type Li_2_FeCl_4_ without (top) and with (bottom) cation ordering of Fe­(II)- and Li-ions on the octahedral sites (C) and the corresponding crystal structures of c-LFC (D) and o-LFC (E).

Having confirmed the spinel motif of o-LFC, we subsequently refined the model of cation-ordered spinel-type o-LFC with Li-ions on the tetrahedral sites against SXRD patterns and found them in good agreement (*R*_w_ = 8.48%, *R*_F_ = 2.11%, Figure 2C, Table S7). Noticeably, the order–disorder phase transition may be suppressed entirely, even at moderate cooling rates of 2 °C min^–1^, resulting in c-LFC at RT (*R*_w_ = 5.99%, *R*_F_ = 2.16%, Figure 2C, Table S8), typical for phase transitions associated with cation ordering.

## Transport Properties of Li_2–*x*_FeCl_4_

Having confirmed the spinel motif of both o-LFC and c-LFC, we studied the Li-ion mobility within the percolating network next. Highly mobile Li-ions require a shallow free energy landscape, in which all sites along the migration pathway exhibit similar energies, and low activation barriers between them.^6,7^ Previous studies on inverse spinel-type Li_2_TMCl_4_ suggest that the more mobile Li-ion resides on the tetrahedral site, and not on the octahedral site shared with the TM, and hopping is facilitated by the energetically accessible face-sharing vacant octahedral site (Figure 3A).^29,44,45^ Indeed, sequential Rietveld refinement of c-LFC upon heating clearly indicated that the Li-ion from the tetrahedral 8a site gradually migrates into the vacant octahedral 16c site (Figure S3G–I, Methods), confirming both its high mobility as well as the 16c site as the energetically accessible intermediate site along the migration pathway.

**Figure 3:**
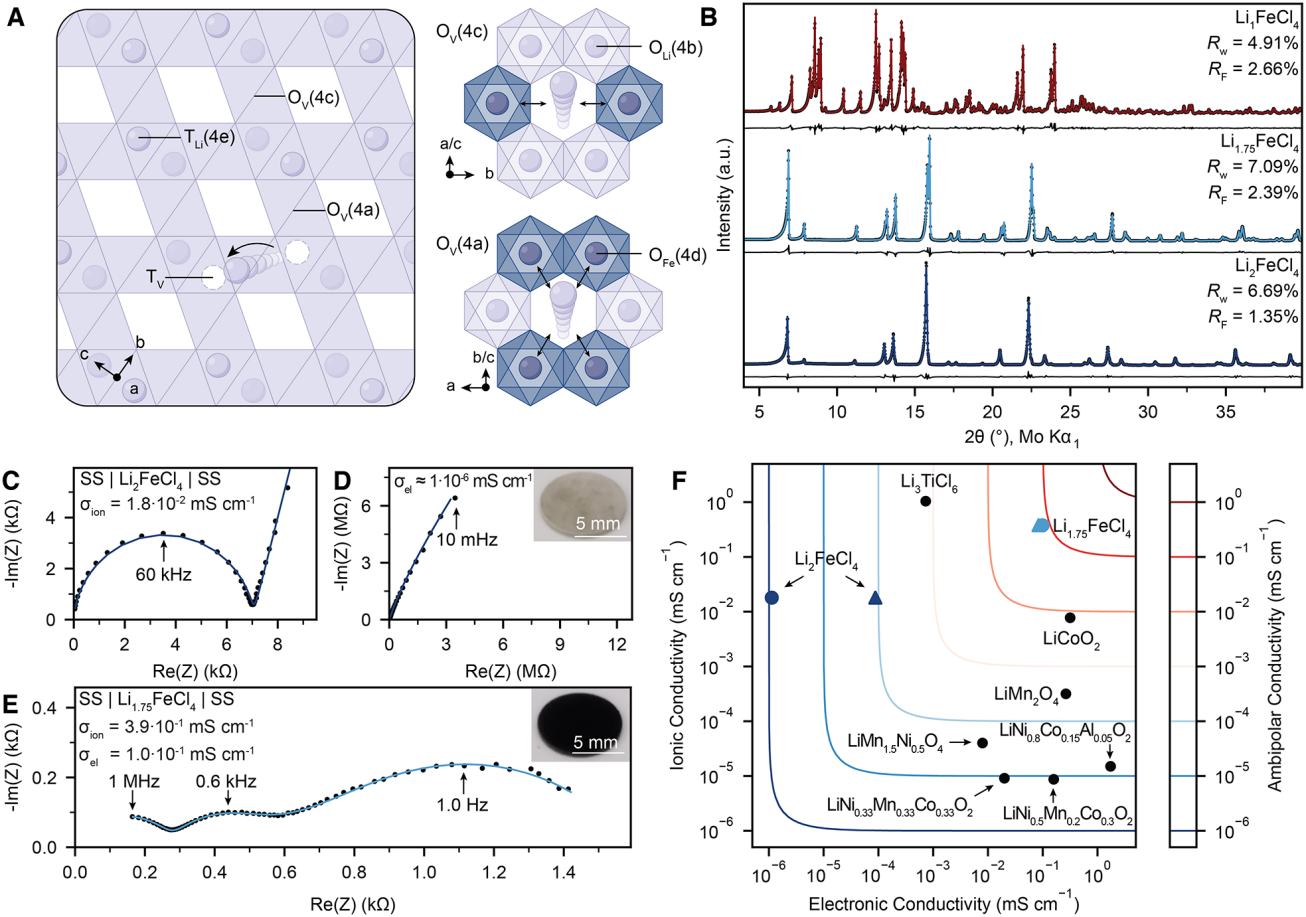
Transport properties and phase diagram of Li_2–*x*_FeCl_4_. (A) Schematic representation of the Li-ion diffusion network in cation-ordered spinel-type o-Li_2_FeCl_4_ and coordination environment of the vacant octahedral 4a and 4c sites. (B) Rietveld refinements for thermally annealed Li_2–*x*_FeCl_4_ (0 ≤ *x* ≤ 1) samples at different Lithium contents. (C–E) Nyquist diagram of the ionic conductivity at 30 °C of spinel-type Li_2_FeCl_4_ (C, D) and spinel-type Li_1.75_FeCl_4_ (E) measured between stainless steel (SS) current collectors. (F) Comparison of transport properties for various Li_*x*_TMO_2_ and Li_*x*_TMCl_4_ CAMs.^33–39,46^ The electronic conductivity of Li_2–*x*_FeCl_4_ was determined from EIS fitting (circles) or LSV (triangles) (Methods, Supplementary Text 3).

The same 3D percolating Li-ion diffusion network characteristic of spinels is preserved in the o-LFC (Figure 3A). Yet, cation ordering of Fe­(II)- and Li-ion induces a splitting of the vacant octahedral site (16c in c-LFC) into two energetically nonequivalent sites (4a and 4c in o-LFC) with different coordination environments (Figure 3A). While the 4c site is coordinated by 2 Fe­(II)- and 4 Li-ions, the 4a site is coordinated by 4 Fe­(II)- and 2 Li-ions. We hypothesize that the lower Coulombic repulsion for migration via the 4c sites biases Li-ions toward migration parallel to the Fe­(II)-ion chains. Yet, the preserved 3D network makes the structure resistant against defects that may block Li-ion diffusion along 1D channels. We note that the degree to which cation ordering occurs in o-LFC will strongly affect Li-ion mobility within o-LFC, and higher conductivities may be achieved by introducing cation disorder.

In apparent contradiction to its fast rate capability,^21,25^ o-LFC displayed moderate ionic conductivity (1.8 × 10^–2^ mS cm^–1^) and poor electronic conductivity (1.1 × 10^–6^ mS cm^–1^), which may be explained by low charge carrier concentrations (Figure 3, Table S9, Supplementary Text 3).^21,25^ Since concomitant Li-ion removal and Fe­(II) oxidation in o-Li_2_FeCl_4_ may significantly increase both ionic and electronic carrier concentrations (Figure 1A), we hypothesized the existence of a highly conductive phase that is formed upon partial delithiation to reconcile the fast rate capability.

We therefore synthesized various compositions within the Li_2–*x*_FeCl_4_ (0 ≤ *x* ≤ 1) phase diagram mechanochemically, followed by subsequent thermal annealing (Methods). Rietveld refinement indicated the presence of three distinct phases at RT in the phase diagram (Li_2_FeCl_4_, Li_1.75_FeCl_4_ and Li_1_FeCl_4_) with negligible solubility ranges between them (Figures 3B, S6, Supplementary Text 4). While full chemical delithiation led to the formation of LiAlCl_4_-type Li_1_FeCl_4_ with a h.c.p. Cl-ion sublattice and Fe­(III) in the tetrahedral sites (Figure S6A,B, Table S14),^47^ o-Li_1.75_FeCl_4_ adopts the same cation-ordered orthorhombically distorted spinel phase as o-Li_2_FeCl_4_ (Figures 3B and S6 and Table S12).

o-Li_1.75_FeCl_4_ was obtained as a black powder, with an electronic conductivity (0.10 mS cm^–1^) 4 orders of magnitude higher than o-Li_2_FeCl_4_ (Figure 3, Table S9, Supplementary Text 3), reflecting the greatly enhanced polaron concentration in o-Li_1.75_FeCl_4_. Similarly, the ionic conductivity was enhanced by more than 20 times (0.39 mS cm^–1^), most likely due to increased Li-ion vacancy concentrations. In this context, the exceptionally high ambipolar conductivity of o-Li_1.75_FeCl_4_ positions it among the most compelling CAMs to date, providing significantly higher electronic conductivity than commercial LiFePO_4_ (LFP) and substantially superior ionic conductivity compared to Li_*x*_TMO_2_ CAMs (Figure 3G).^33–39^

## Lithiation Mechanism of Li_2_FeCl_4_

Although previously disregarded due to their high solubility in liquid electrolytes,^48^ lithium metal chlorides, e.g., LFC may be explored as CAMs in all-solid-state Li-metal batteries (ASSBs) with the advent of oxidatively stable solid-state electrolytes (SSEs).^49–51^

The absence of any appreciable solid-solution behavior for spinel-type Li_2_FeCl_4_, spinel-type Li_1.75_FeCl_4_ or LiAlCl_4_-type Li_1_FeCl_4_ has important consequences for the prospective electrochemical delithiation mechanism. While Li_2_FeCl_4_ and Li_1.75_FeCl_4_ share a close structural relationship that may facilitate a rapid two-phase reaction, transforming into Li_1_FeCl_4_ would entail a reconstructive phase transformation involving complete cation and anion sublattice rearrangement. Moreover, solid-solution processes, if present, must be kinetically driven rather than thermodynamically driven.

To investigate the (de)­lithiation mechanism of Li_2_FeCl_4_, and the potential involvement of a transient Li_1.75_FeCl_4_ phase, cathodes comprising Li_2_FeCl_4_, Li_3_YCl_6_ (LYC) SSE and carbon black (CB) were fabricated and pressed onto LYC pellets, using LiIn as the counter and reference electrodes (Methods). LYC was chosen as the SSE as opposed to the more highly conductive Li_6_PS_5_Cl because of its (electro)­chemical incompatibility with the CAM (Figure S7). Galvanostatic cycling was performed with an upper cutoff voltage of 3.4 V vs LiIn to prevent irreversible Cl^–^ oxidation observed at higher potentials (Figure S8).

To probe the (de)­lithiation mechanism, including a potential delithiation-induced cation ordering in Li_2_FeCl_4_, cathodes containing c-LFC were studied by *operando* SXRD during galvanostatic cycling in a specially designed cell (Figure S10, Methods). Since the X-rays penetrate the entire cell, the dominant signal contribution from the LYC SSE was subtracted to isolate the Li_2–*x*_FeCl_4_ signal (Figure 4A,B, Methods).

**Figure 4:**
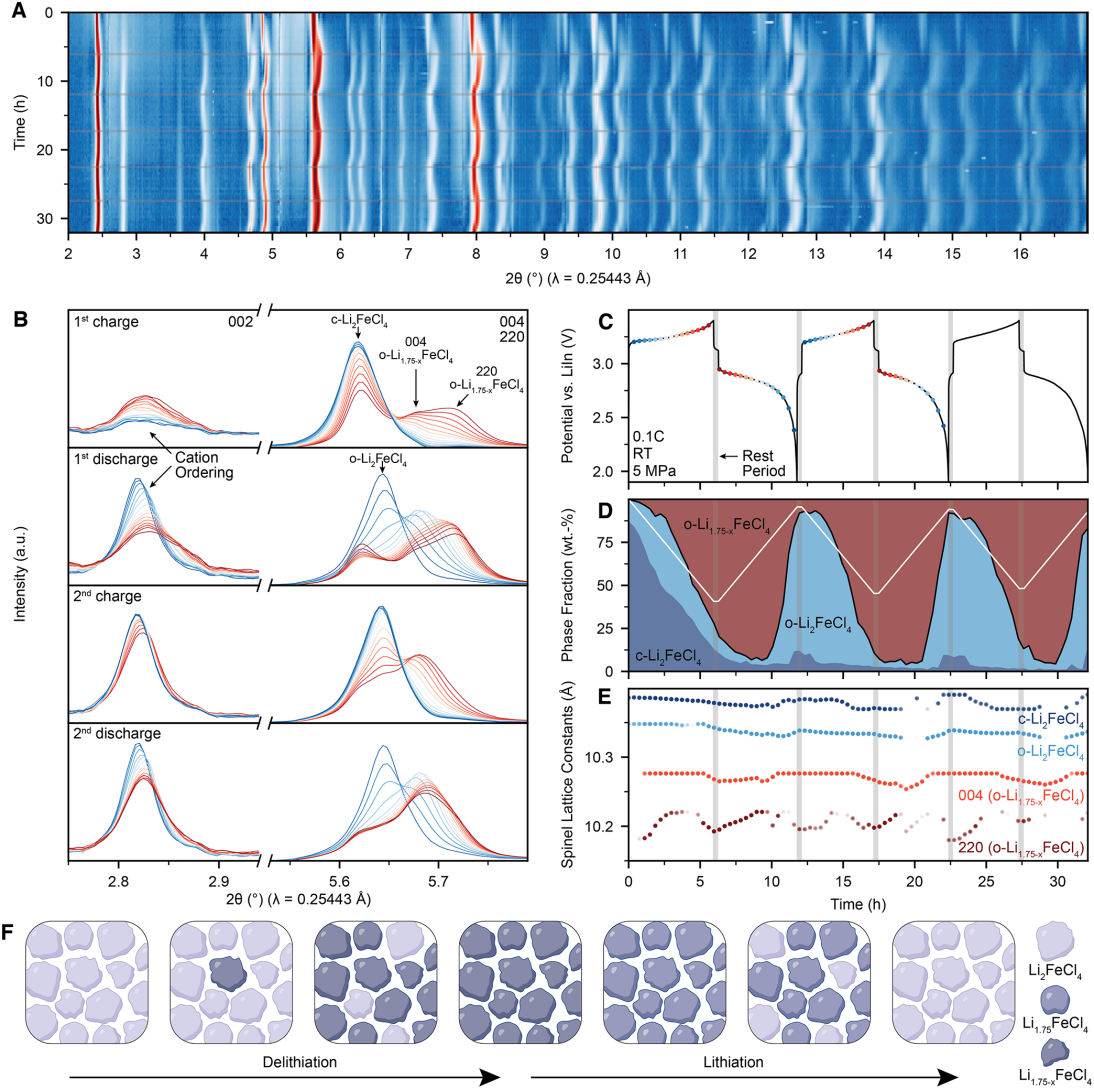
Lithiation mechanism of Li_2_FeCl_4_. (A) *In-situ* SXRD spectra of all-solid-state batteries with cathodes containing Li_2_FeCl_4_. A logarithmic intensity scale was used to enhance low-intensity reflections. Gray areas indicate rest periods in the galvanostatic cycling profile. (B) SXRD profile of the 002 and 004/220 reflections during the 1st and 2nd cycle. (C) Voltage profile of the all-solid-state battery (black line) with the points of XRD acquisition highlighted (blue/red dots). (D) Evolution of phase fractions obtained from peak fitting of the 004/220 reflection. The white line indicates the linear evolution expected for galvanostatic cycling for a hypothetical two-phase reaction between Li_2_FeCl_4_ and Li_1_FeCl_4_. (E) Evolution of cell parameters of the phases expressed in the spinel unit cell obtained from the peak fitting of the 004/220 reflection. Light points indicate points with larger errors. Data points with relative errors larger than 1% were omitted for clarity. (F) Proposed asymmetric lithiation and delithiation mechanism of spinel-type Li_2–*x*_FeCl_4_.

During charging, reflections corresponding to c-LFC gradually vanish, while a new set of reflections, including the superstructure reflections associated with cation ordering, emerge at higher angles (Figure 4, A and B). This evolution indicates a two-phase delithiation process, akin to LFP,^52,53^ between a cation-disordered lithiated spinel (c-LFC) and a cation-ordered delithiated spinel (o-Li_1.75–*x*_FeCl_4_). We note that a small fraction of c-LFC persists after charging, likely due to incomplete utilization of the CAM, reflecting the suboptimal cell design, as also suggested by the limited charge capacity.

The delithiated spinel phase exhibits peak splitting of the 004 and 220 reflections, in line with the distorted o-Li_1.75–*x*_FeCl_4_ phase (Supplementary Text 4). Interestingly, LiAlCl_4_-type Li_1_FeCl_4_ is not observed upon delithiation, suggesting a large solid-solution range for o-Li_1.75–*x*_FeCl_4_. Consistent with the solid-solution behavior of o-Li_1.75–*x*_FeCl_4_, the 220 reflection shifts to higher angles and further away from the 004 reflection upon charging, marking a contraction in the *ab*-plane as a result of continued Li^+^ extraction and Fe^2+^ oxidation. The formation of metastable o-Li_1.75–*x*_FeCl_4_ is likely observed due to the large activation energy required to form the thermodynamically stable Li_1_FeCl_4_ phase. During discharge, the 004 and 220 reflections move closer together again, indicating a reversible solid-solution behavior. Meanwhile, the increased 002 reflection intensity indicates increased cation ordering in o-Li_1.75–*x*_FeCl_4_.

Upon discharge (*ca*. 10 h), the continued decrease of reflections associated with o-Li_1.75–*x*_FeCl_4_, concomitant with the simultaneous emergence of a set of reflections at lower angles, marks the interconversion of o-Li_1.75–*x*_FeCl_4_ into a lithiated spinel phase. The continued presence of the 002 reflection indicates that the newly formed lithiated spinel is cation-ordered (o-Li_2_FeCl_4_). This suggests that beyond the reversible two-phase reaction between Li_2_FeCl_4_ and Li_1.75–*x*_FeCl_4_, and the solid-solution behavior within Li_1.75–*x*_FeCl_4_, electrochemical cycling also induces cation ordering, as evidenced by the formation of cation-ordered spinels (o-Li_2_FeCl_4_ and o-Li_1.75–*x*_FeCl_4_) from a disordered precursor (c-LFC). Their persistence in subsequent cycles highlights their stability at RT over cation-disordered spinels.

To further quantify the (de)­lithiation kinetics, reflections whose intensity is insensitive to cation ordering or Li^+^ content (e.g., the 220/004, the 044/404 etc.) were analyzed to track the respective spinel phase fractions and unit cell parameters (Methods, Figure 4D,E, Figure S13). During the first charge, Li_2_FeCl_4_ (black line) gradually disappears while o-Li_1.75–*x*_FeCl_4_ is formed, reflecting a two-phase reaction during charge. However, the respective phase fractions do not change symmetrically with respect to delithiation and lithiation and are not in accordance with an idealized two-phase reaction between Li_2_FeCl_4_ and a hypothetical Li_1_FeCl_4_ phase (white line). Instead, the Li_2_FeCl_4_/o-Li_1.75–*x*_FeCl_4_ phase fractions remain constant throughout much of the lithiation, raising the question of how Li-ions are stored if the phase fractions stay constant and only rapidly change at the end delithiation. Instead, o-Li_1.75–*x*_FeCl_4_ must be lithiated within the same phase by a solid-solution mechanism, which is further evidenced by the pronounced peak shifts of the 220 reflection during discharge (Figure 4B,E).

The asymmetric charge and discharge kinetics of Li_2–*x*_FeCl_4_ can be attributed to the different transport properties of Li_1.75–*x*_FeCl_4_ compared to those of Li_2_FeCl_4_ (Figure 4F). Upon initial formation of a marginally small o-Li_1.75_FeCl_4_ phase fraction via a two-phase reaction, its ambipolar conductivity is several orders of magnitude higher than that for the remaining Li_2_FeCl_4_, while its delithiation potential is almost identical. o-Li_1.75_FeCl_4_ will therefore be subsequently delithiated via a solid-solution mechanism to form metastable Li_1.75–*x*_FeCl_4_, rather than the competing two-phase reaction of the remaining Li_2_FeCl_4_. We note that most likely, the solid-solution and two-phase reaction do not compete on a single-particle level but rather on a cathode level. Only once the existing fraction of Li_1.75–*x*_FeCl_4_ is fully delithiated does the slower two-phase reaction of Li_2_FeCl_4_ proceed until the entire cathode is delithiated. During discharge, the highly electronically and ionically conductive Li_1.75–*x*_FeCl_4_ phase is rapidly lithiated to Li_1.75_FeCl_4_, before its kinetically slower two-phase reaction toward Li_2_FeCl_4_ proceeds. Such asymmetric charge–discharge kinetics have also been observed in other Li_*x*_TMO_2_ CAMs.^54,55^

## Electrochemical Performance of Li_2_FeCl_4_ in All-Solid-State Batteries

The superior ductility of chlorides compared to oxides offers distinct advantages in ASSBs, including intimate contact with the SSE, and resistance to strain-induced CAM particle fracture.^46,56^ Additionally, the existence of the highly electronically and ionically conductive Li_1.75–*x*_FeCl_4_ phase over a wide degree of lithiation may enable ASSBs with very high rate capability and cycling stability. To isolate the intrinsic charge–discharge properties, initial ASSBs employed low-mass loading electrodes with high SSE and CB contents to eliminate any extrinsic ionic and electronic transport limitations.^57^ As the highly conductive Li_1.75–*x*_FeCl_4_ phase forms immediately upon charging, cathode nanostructuring is not required, unlike in LFP,^58^ and the employed LFC particles were 1–10 μm large, comparable to commercial Li_*x*_TMO_2_ CAMs (Figure 5A). An initial charge and discharge capacity of 121.7 mA h g^–1^ and 117.4 mA h g^–1^, respectively was observed at 0.1C (1C = 127 mA h g^–1^) (Figure 5B,C). While the capacity decays slightly in subsequent cycles, the rate capability remained largely unaffected up to 1C with charge and discharge capacities of 106.5 and 106.2 mA h g^–1^, respectively. At higher rates (2C and 5C), a moderate capacity drop occurred, in line with increased polarization during the end of discharge, when Li_1.75_FeCl_4_ reacts back to Li_2_FeCl_4_. However, the capacity was fully recovered upon returning to 1C, and the cell demonstrated very high cycling stability at 0.1C.

**Figure 5:**
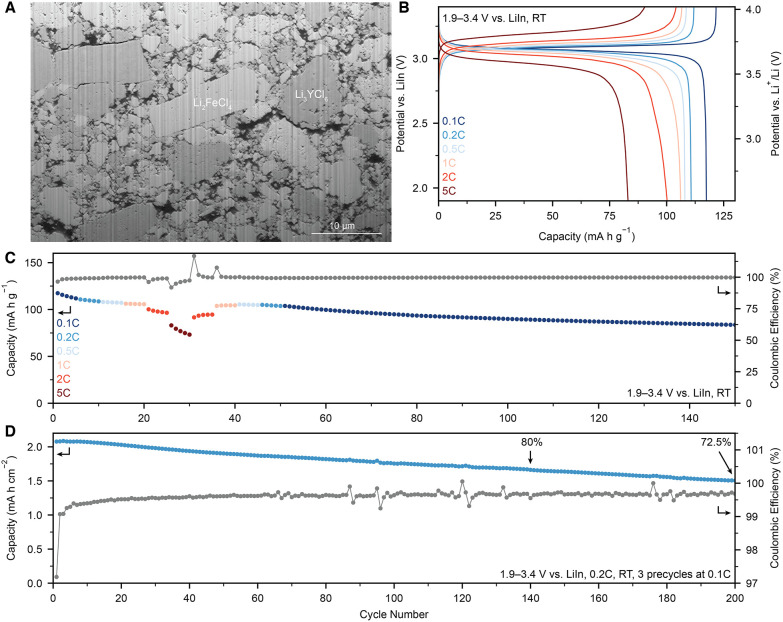
Electrochemical performance of Li_2_FeCl_4_. (A) Cross-sectional SEM micrographs of pristine Li_2_FeCl_4_ cathodes. (B, C) Voltage profile (B) and cycling stability (C) of all-solid-state batteries with cathodes containing Li_2_FeCl_4_ at different rates. (D) Cycling stability of the all-solid-state batteries with high areal loading and high CAM fraction (70 wt %) of Li_2_FeCl_4_.

To further corroborate the involvement of Li_1.75_FeCl_4_ in the (de)­lithiation of Li_2_FeCl_4_, cathodes containing Li_1.75_FeCl_4_ were also cycled (Figure S14). The ASSBs demonstrated very similar voltage profiles compared to Li_2_FeCl_4_, and high rate capability up to 5C.

Encouraged by these results, we fabricated high areal-capacity cells (70 wt %_LFC_, 21.5 mg_LFC_ cm^–2^). To ensure the formation of a fully cation-ordered spinel, three precycles were performed at a current density of 0.27 mA cm^–2^ (Figure S15). The ASSB was then cycled at 0.5 mA cm^–2^ (0.2C), delivering an exceptionally high initial charge and discharge capacity of 2.1 mA h cm^–2^ at 97.2% Coulombic efficiency (CE) (Figure 5D). By the second cycle, the CE increased to 99.1%, and stabilized at 99.7% in subsequent cycles, resulting in a capacity retention of 80% (1.66 mA h cm^–2^) and 72.5% (1.51 mA h cm^–2^) after 140 and 200 cycles, respectively.

Although further work is needed to address the poor moisture stability of halospinel CAMs, our results, in conjunction with previous work, unlock TMs with a preferred +II/+III redox cycle, such as earth-abundant Fe, for use in spinel-type CAMs. When partially delithiated, halospinels demonstrate significantly higher ionic conductivity than oxospinels while inheriting their high electronic conductivity. The highly conductive o-Li_1.75–*x*_FeCl_4_ phase enables chemical diffusion more than 10 times higher than for the fastest state-of-the-art CAM, and ensures superior charge transport throughout cycling, resulting in high-rate capability and high areal capacity. Integration of halospinels as a redox-active conductive catholyte with state-of-the-art oxide cathodes offers a promising pathway to further enhance the overall energy density.

## Supplementary Material



## Data Availability

All data are available in the main text or the Supporting Information and available from the corresponding author upon reasonable request.
