# Supplementary material for: A Highly Conductive Halospinel Cathode for All-Solid-State Batteries
Source: ACS Energy Lett. 2025 Oct 31;10(11):5891–9. doi: 10.1021/acsenergylett.5c02476 (PMC12624834; doi:10.1021/acsenergylett.5c02476)
Supplement: Supplementary file 1 [file nz5c02476_si_001.pdf]

Supplementary Materials for

A Highly Conductive Halospinel Cathode for All-Solid-State Batteries

Julian F. Baumgärtner,[1,2] Daniel Isler,[1,2] Hung Quoc Nguyen,[3] Matthias Klimpel,[1,2] Jaka Šivavec,[1,2] Chris Černe,[1,2] Dmitry Chernyshov,[4] Wouter van Beek,[4] Daniel Rettenwander,[3,5,6] Kostiantyn V. Kravchyk,[1,2] and Maksym V. Kovalenko[1,2*]*

[1]Laboratory of Inorganic Chemistry, Department of Chemistry and Applied Biosciences, ETH Zürich, CH-8093 Zürich, Switzerland.

[2]Laboratory for Thin Films and Photovoltaics, Empa - Swiss Federal Laboratories for Materials Science & Technology, CH-8600 Dübendorf, Switzerland.

[3]Department of Material Science and Engineering, NTNU Norwegian University of Science and Technology, 7034 Trondheim, Norway.

[4]Swiss–Norwegian Beam Lines at the European Synchrotron Radiation Facility, 38000 Grenoble, France.

[5]Christian Doppler Laboratory for Solid-State Batteries, NTNU Norwegian University of Science and Technology, 7034 Trondheim, Norway.

[6]Austrian Institute of Technology GmbH, Center for Transport Technologies, Battery Technologies, Vienna 1210, Austria

*Corresponding authors. Emails: Kostiantyn.Kravchyk@empa.ch and mvkovalenko@ethz.ch

MATERIALS

The preparation of all compounds was carried out under Ar atmosphere.

Mechanochemical Synthesis of $Li_{2-x}FeCl_4$

The mechanochemical synthesis of all $Li_{2-x}FeCl_4$ (x= 0, 0.125, 0.25, 0.5, 1) samples was adapted based on a previous report.[1] $FeCl_2$ (99.5%, abcr) and $FeCl_3$ (99.9%, Sigma-Aldrich) were used as received. LiCl (99.9%, Thermo Scientific) was dried at 80 °C under vacuum over night. 1 g batches were prepared with the stoichiometric amounts and preground in an agate mortar for 5 – 10 min, before transferring into a ZrO_2 beaker (20 mL) with ZrO_2 balls (20 g, 3 mm Ø), which were subsequently sealed air-tight with sealing foil. The mechanochemical reaction was carried out in a planetary ball-mill (Fritsch, Pulverisette 7, Classic Line) for 20 cycles using two steps of 10 cycles each, with a cycle consisting of a milling time of 15 min at 500 rpm counterclockwise, followed by a rest step for 5 min, then 15 min at 500 rpm clockwise rotation, followed by a rest step for 5 min. After each step, the mixture was taken into the glovebox and homogenized, in line with previous reports.[2] Li_2FeCl_4, $Li_{1.75}FeCl_4$ and Li_1FeCl_4 were obtained as light-beige, black, and dark orange powders respectively.

Heat Treatment of $Li_{2-x}FeCl_4$

The ball-milled $Li_{2-x}FeCl_4$ (x= 0, 0.125, 0.25, 0.5, 1) powder (*ca.* 70 mg) was loaded into a pressing die (8 mm Ø) and uniaxially compressed into dense pellets at 2 t (390 MPa) for 3 min. The pellets were then placed in stainless steel coin cells and heated on a preheated hot plate. Li_2FeCl_4 was heated at 300 °C for 5 min. c-LFC was obtained by rapidly removing the pellet from the coin cell to quench the high-temperature phase. o-LFC was obtained by subsequently heating it on a hot plate at 120 °C for 72 h. Compounds containing $FeCl_3$ readily sublimate and decompose

above 120 °C.[3] $Li_{2-x}FeCl_4$ (x= 0.125, 0.25, 0.5, 1) samples were therefore thermally annealed at 100 °C for 1 h, before letting the sample cool down on the hot plate at a natural cooling rate.

Mechanochemical Synthesis of Li_3YCl_6

The mechanochemical synthesis of LYC was adapted based on a previous report.[2] LiCl (99.9%, Thermo Scientific) and YCl_3 (99.99%, Sigma-Aldrich) were used as received. 0.66 g batches were prepared with 10wt.-% excess of YCl_3 and preground in an agate mortar for 10 min, before transferring into a ZrO_2 beaker (20 mL) with ZrO_2 balls (20 g, 3 mm Ø), which were subsequently sealed air-tight with sealing foil. The mechanochemical reaction was carried out in a planetary ball-mill (Fritsch, Pulverisette 7, Classic Line) for 120 cycles using four steps of 30 cycles each, with a cycle consisting of a milling time of 15 min at 600 rpm counterclockwise, followed by a rest step for 5 min, then 15 min at 600 rpm clockwise rotation, followed by a rest step for 5 min. After each step, the mixture was taken into the glovebox and homogenized. LYC was obtained as a white powder.

METHODS

Powder X-Ray Diffraction

Powder XRD patterns were collected at RT on a Stoe STADI P powder X-ray diffractometer (Mo Kα1 radiation, $\lambda = 0.709300$ Å, focusing germanium monochromator) equipped with a Dectris Mythen 1 K silicon strip detector. Samples were prepared in a 0.5 mm Ø borosilicate glass capillary and sealed under Ar and measured in Debye-Scherrer geometry.

Synchrotron X-Ray Diffraction

SXRD data were obtained at the BM01 and BM31 beamline at the European Synchrotron Radiation Facility. At BM01, the X-ray beam (λ = 0.71796Å or 0.72538 Å) was tuned with a sagitally focusing Si(111) double crystal monochromator and a set of collimating and vertically Rh-coated Si mirrors to a final beam size of *ca*. 0.1 mm x 0.35 mm. Diffraction patterns were collected in transmission mode on a Pilatus 2M area photon-counting detectors, and azimuthally integrated using BUBBLE.[4] Patterns were acquired in a 2θ range of 0.5 – 50 ° with a step size of 0.0025 ° at room temperature (RT). Samples were prepared in a 0.5 mm Ø borosilicate glass capillary and sealed under Ar. For the variable-temperature SXRD at BM01, the sample was heated under air and cooled down again at various rates using a Cryostream 700+.

At BM31, the X-ray beam (λ = 0.25448 Å) was monochromatized using a liquid nitrogen double crystal monochromator equipped with a flat and a fixed radius sagital Si(111)crystal. Diffraction patterns were collected in transmission mode on a CdTe Pilatus 2M area photon-counting detectors, and azimuthally integrated using BUBBLE.[4] Patterns were acquired for 20 s in a 2θ range of 0.5 – 20 °with a step size of 0.004 ° at RT.

Rietveld Refinement

Rietveld refinement was performed with the GSAS-II program.[5] Instrumental parameters were determined with a LaB_6 NIST standard prepared with the same setup that was used for real measurements. The background was modelled by a Chebyschev inverse polynomial The refined values are summarized in Tables S1-S8, and S10-S14.

Sequential refinement of c-Li_2FeCl_4 during *in-situ* heating was performed using every fifth acquired pattern (17 in total). The background was again modelled by a Chebyschev polynomial with 30 coefficients. Crystallite size and microstrain of c-Li_2FeCl_4 were directly refined, while the lattice parameters were optimized using elastic strain relative to the initial lattice parameters. To model the Li-ion occupancy of the 8a and 16c site, two c-Li_2FeCl_4 phases were used with pure 8a or 16c occupancy respectively. Since the scattering intensity of Li-ions is weak, the refined occupancies are not expected to be exact. Nevertheless, trends can be extracted from sequential refinement of successive SXRD patterns. All refinable parameters between the two c-Li_2FeCl_4 phases were constrained to be identical, and only their respective phase fractions were varied to obtain the occupancy. All other parameters were kept constant.

Background Subtraction of in-situ SXRD During Electrochemical Cycling

To isolate the signal contribution from the $Li_{2-x}FeCl_4$ CAM from the dominant LYC solid state electrolyte (SSE) contribution, a background subtraction was performed with Python using scipy. As the LYC SSE does not undergo a chemical reaction, its signal contribution does not vary with time, whereas the signal contribution from the $Li_{2-x}FeCl_4$ CAM is time-dependent, it can be isolated by differentiating the SXRD with respect to time, and reintegrating it $\left(I_{\mathrm{bkg}} = \int \frac{\partial I(2\theta, t)}{\partial t} dt + C(2\theta)\right)$ (Figure S11). Given that reflection intensity cannot be negative, the

reintegration constant needs to be chosen so that $C(2\theta) \geq 0$. The background obtained by this method removes all signal contributions from the SXRD that do not vary with time, which may include residual signal contribution of $Li_{2-x}FeCl_4$, if a part of the $Li_{2-x}FeCl_4$ reflection does not vary with time. To account for this, I_{bkg} was interpolated with a polynomial in the regions of $Li_{2-x}FeCl_4$ reflections (Figure S12). Using a polynomial to interpolate in ranges of $Li_{2-x}FeCl_4$ reflections also reintegrates overlapping LYC SSE reflections. These contributions were subtracted subsequently by fitting the LYC peak contribution assuming a Pearson VII profile function, and adding them to I_{bkg} (Figure S12).

Phase Analysis of in-situ SXRD During Electrochemical Cycling

To quantify the phase fractions of c-Li_2FeCl_4, o-Li_2FeCl_4 and o-$Li_{1.75-x}FeCl_4$, their relative peak intensities for a shared set of reflections were quantified. Certain reflections (e.g. the 220/004, the 044/404 etc.) are neither influenced by the degree of cation ordering, nor by the Li content within a given spinel phase, so their relative areas are directly proportional to the respective weight fractions, while the peak position can be used to extract the unit cell parameters (Figure S13). Peak profile fitting was performed using Pearson VII functions. For a better comparison of the unit cell parameters of both cubic and orthorhombic phases, the lattice constants for the orthorhombic modifications ($x_{\mathrm{o}}, x \in a, b, c$) were related back to the cubic spinel structure (x_{c}), according to $a_{\mathrm{c}} = b_{\mathrm{c}} = c_{\mathrm{c}} = c_{\mathrm{o}} \approx \sqrt{2}\, b_{\mathrm{o}} \approx \sqrt{2}\, a_{\mathrm{o}}$.

Focused Ion Beam Scanning Electron Microscopy

Cross-section FIB-SEM images were acquired on a Thermo Fisher Scientific Helios 5 Hydra Multi-Ion-Species Plasma FIB Microscope using Xenon as working gas. To obtain a smooth

cross-section, a carbon pad with a thickness of 5 μm was deposited on the surface using anthracene via the gas injection system, with an ion beam acceleration voltage of 12 kV and a current of 20 nA. Cross-sections were prepared by Xenon ion beam milling with an acceleration voltage of 30 kV and a current of up to 2.5 μA. Corresponding SEM images were recorded on the circular backscatter detector (5 kV acceleration voltage, 1.6 nA beam current, 4.1 mm working distance, 52 ° stage tilt). Imaging artifacts, i.e. curtaining from the ion beam milling, were removed using the VSNR v2 plugin in ImageJ Fiji software.[6, 7]

Preparation of Li_2FeCl_4 and $Li_{1.75}FeCl_4$ All-Solid-State Cathodes

All steps for ASSB preparation were carried out under Ar atmosphere. For cathode preparation, a coarse-grained powder of heat-treated c-LFC or heat-treated o-$Li_{1.75}FeCl_4$ (30 – 50 mg) was transferred into a ZrO_2 bowl (10 mL) with ZrO_2 balls (3 g, 3 mm Ø), and homogenized in a frequency ball mill (Fritsch, Pulverisette 23 Mini) for 2 cycles with a shaking time of 1 min per cycle at 30 Hz. Between the cycles, the bowl was rotated by 180°. Into the same bowl, carbon black (CB, Super C65, TIMCAL) and LYC were added in the desired weight fractions for a total cathode composite mass of 70 mg (*vide infra*). The cathode components were then homogenized for 2 cycles with a shaking time of 2 min per cycle at 15 Hz. Between the cycles, the bowl was flipped by 180°. The SSE was added to improve the ionic conductivity of the fully lithiated composite cathode.

Low-mass loading electrodes were prepared by mixing c-LFC (28 mg, 40 wt.-%), LYC (38.5 mg, 55 wt.-%) and CB (3.5 mg, 5 wt.-%), and cathode loadings were 6-7 mg cm^{-2} (*ca.* 2.5 mg cm^{-2} c-LFC). High-mass loading electrodes were prepared by mixing c-LFC (49 mg, 70 wt.-%), LYC (18.9 mg, 27 wt.-%) and CB (2.1 mg, 3 wt.-%), and cathodes loadings were *ca.* 30 mg cm^{-2} (*ca.* 21 – 22 mg cm^{-2} c-LFC).

Preparation and Assembly of All-Solid-State Cells

All-solid-state batteries were prepared by loading LYC powder (30 – 40 mg) into a pressing die (8 mm Ø) and uniaxially compressing it at 0.15 t (30 MPa) for 10 s to create a flat surface. Afterwards, the cathode composite was added on one side of the formed LYC pellet and pressed at 2 t (390 MPa) for 3 min. Then an Li disc (60 μm thick, 8 mm Ø) was cut out and rolled from Li rods (Sigma-Aldrich, 99.9%, 12.7 mm Ø) and sandwiched between two In discs (Goodfellow, 99.999%, 100 μm thick, 8 mm Ø) and attached to the other side of the pellet cell. To allow for LiIn alloy formation, the assembled cell was placed into a solid-state cell holder (Sphere, ASC-A+), and pressurized at 75 MPa for 3 min, followed by 50 MPa for 2 h. After this, the cell was removed and placed into a solid-state cell holder (Sphere) and screwed tight with a hex key.

Preparation and Assembly of All-Solid-State Cell for Operando SXRD

The operando SXRD experiments were performed in a custom-made pressure cell and the X-ray beam was shot perpendicular through the whole cell. Two glassy carbon windows (Almath Crucibles) with a thickness of 0.5 mm each were used as a beam-transparent window. A constant pressure was applied in a feedback loop. In the first step, the pressure was determined using a calibrated, ring-shaped load cell (Zhimin ZMMC4) in combination with a load cell amplifier (SparkFun HX711) and an Arduino Uno R4 Minima. In the second step, the pressure was adjusted using a ring-shaped Piezo stack (Thorlabs PK44M3B8P2) and a piezo controller (Thorlabs MDT693B).

Electrochemical Measurements

All cells were measured inside a glove box at 28 – 30 °C. Electrochemical impedance spectroscopy (EIS) measurements were conducted on a BioLogic SAS MTZ-35 impedance analyzer. Linear sweep voltammetry (LSV), cyclic voltammetry and galvanostatic cycling measurements were performed on a multichannel potentiostat/galvanostat from Biologic (MPG2). Potentiostatic EIS of LFC pellets was conducted in the solid-state cell holder between two stainless steel electrodes that were polished mirror-like to provide flat contact area. EIS was conducted in a frequency range of 1 MHz to 10 mHz with a sinus peak amplitude of 10 mV. LSV of SS|LFC|SS pellets was performed in a voltage range of 0 – 100 mV at slow rates (2 $\mu V\ s^{-1}$) to allow full equilibration. More details on the EIS and LSV measurements can be found in the Supplementary Text, section 3. Cyclic voltammetry of ASSBs containing LFC cathodes was performed in a voltage range between 1.7 – 4.7 V vs. LiIn at 0.2 $mV\ s^{-1}$. Galvanostatic cycling of ASSBs containing LFC cathodes was performed in a voltage range between 1.9 – 3.4 V vs. LiIn. C-rates were calculated with respect to the theoretical capacity for one-electron oxidation of Li_2FeCl_4 (127 $mA\ h\ g^{-1}$).

Electrochemical Impedance Spectroscopy Modelling

Experimental EIS spectra were fitted using a custom python framework. The Jamnik-Maier transmission line (TML) model was used to fit the experimental EIS spectra,[8, 9] with the analytical impedance expression provided by Lai and Haile (Supplementary text, section 3, Figure S4a).[10] Capacitors were modelled by constant phase elements (CPEs) to account for non-ideal behavior. Model parameters were fitted to the complex impedance data using complex nonlinear least squares optimization,[11, 12] with the Trust Region Reflective algorithm provided in scipy 1.13.0. To

ensure that all impedance values contribute equally to the objective function regardless of their magnitude, the residuals were weighted using the reciprocal modulus of the experimental impedance.[11] Parameter uncertainties were estimated from the covariance matrix of the Jacobian, scaled by the residual variance.

SUPPLEMENTARY TEXT

1. Phase Analysis of as-prepared Li_2FeCl_4

To identify the product phases in as-prepared Li_2FeCl_4, SXRD was measured (Figure S1). Since the reflections are significantly broadened due to size and microstrain, significant peak overlap is observed. c-Li_2FeCl_4, o-Li_2FeCl_4, as well as the other reported compounds in the $Li_{2-2x}Fe_{1+x}Cl_4$ phase diagram (LiCl, $FeCl_2$, and Li_6FeCl_8) all share an f.c.c. chloride sublattice and very similar intralayer distances. The XRD reflections of the respective phases are therefore strongly overlapping and may not be discriminated based on simple indexing.

To identify the correct phases and deconvolute their contributions, Rietveld refinement was performed. Refinement with a single o-Li_2FeCl_4 phase yielded similar agreement with either the *Imma* (R_w=7.5%, R_F=2.5%, Figure S1a, Table S1) or *Cmmm* (R_w=7.2%, R_F=2.3%, Figure S1b, Table S2) space group. This highlights the similarity between those two structures when analyzed by powder XRD. However, both models show systematic mismatches in the peak intensity at 14°, 16°, and 24°.

These mismatched intensities indicate the presence of a second phase with strongly overlapping reflections. In line with previous studies, this phase may either be LiCl ($Fm\bar{3}m$) or Li_6FeCl_8 ($Fm\bar{3}m$).[13] While refinements in the presence of an additional LiCl improved the fit significantly (Figure S1c,d, Table S3 and S4), unreasonably large lattice constants (5.16 Å) and microstrains (>1.1%) were obtained. This indicated the presence of Fe(II) inside the rocksalt structure. If Fe(II) was statistically distributed over the octahedral sites, it would be consistent with the apparent LiCl phase. Due to the presence of Fe(II) inside of the structure, it would give an apparent LiCl constant of 5.155 Å (0.6% deviation), agreeing closely with the refined one.

If Fe(II) was ordered on the octahedral sites, it would result in the Li_6FeCl_8 phase, with a unit cell *ca*. twice the size of the original LiCl unit cell, and characterized by additional superstructure reflections,[14] which would strongly overlapping with the reflections of both *Cmmm* and *Imma* phases. Hence, the refinements were repeated in the presence of Li_6FeCl_8. Refinement in the presence of a secondary Li_6FeCl_8 phase also improved the fits for both the *Imma* model (R_w=6.6%, R_F=2.0%, Figure S1e, Table S5) and the *Cmmm* model (R_w=6.4%, R_F=1.9%, Figure S1f, Table S6). Moreover, the refined lattice constant for Li_6FeCl_8 (10.306 Å) agreed closely with the reported value in the literature (10.311 Å, 0.05% deviation). We note that the presence of either LiCl or Li_6FeCl_8 also implies a slightly Li-deficient o-$Li_{2-2x}Fe_{1+x}Cl_4$ phase, in line with the previously determined phase diagram, which was considered when refining the occupancies of the o-$Li_{2-2x}Fe_{1+x}Cl_4$ phase.[13]

It is noteworthy, however, that the fits only improve marginally in the presence of Li_6FeCl_8, relative to phase-pure models, even though the fits including Li_6FeCl_8 use as much as 37 wt.-% of Li_6FeCl_8. This is explained by the complete overlap of the reflections, as all phases are structurally very similar, resulting in highly negatively correlated scale factors (-0.98) between the two phases. In conclusion, this highlights that phase analysis of as-synthesized Li_2FeCl_4 powder based on Rietveld refinement alone may not be conclusive due to the high structural similarity between o-Li_2FeCl_4 and its potential side products, especially LiCl and Li_6FeCl_8, resulting in very strong correlations between the respective scale factors. Phase analysis should therefore be performed on more crystalline samples where peak overlap is reduced.

2. Second-Order Phase Transition

According to Landau theory for phase transitions,[15] phase transitions are characterized by a change of a suitable order parameter η, depending on the thermodynamic state variables, which may be interpreted as a measure of symmetry breaking in the equilibrium state. η vanishes at or above the critical point, e.g. the critical temperature T_C of the phase transition, and attains a non-zero finite value below it. The Landau free energy $G(\eta)$ is a phenomenological expression of the free energy G of a system at a fixed value of η. For continuous (second-order) phase transitions, η is small near the phase transition, allowing $G(\eta)$ to be approximated by the first terms of a Taylor expansion in η, and η will change continuously from zero to a small non-zero value at T_C. Meanwhile, a first-order phase transition will not display a continuous change in $G(\eta)$, and therefore also not of η around the critical point. *Vice versa*, the type of phase transition can be inferred from the temperature evolution of $\eta(T)$. In the classical Landau theory for 2nd order phase transitions, the Taylor expansion of $G(\eta)$ is truncated after the fourth term, giving rise to a characteristic temperature dependence according to equation 1.

$$\eta^2 \propto (T_c - T) \tag{1}$$

To determine the kind of phase transition in Li_2FeCl_4, a suitable order parameter η needs to be determined. While the high temperature phase (c-Li_2FeCl_4) exhibits no preferential ordering of Li- and Fe(II)-ions on the octahedral 16d site, these cations order in the low-temperature phase (o-Li_2FeCl_4). η for cation ordering may therefore be the preferred occupation by one element on the octahedral sites of interest, according to equation 2:

$$\eta = \frac{o_{\mathrm{Fe}} - o_{\mathrm{Li}}}{o_{\mathrm{Fe}} + o_{\mathrm{Li}}} \tag{2}$$

Here, o_j denotes the occupancy of a species j on a given site. η will be 0 in c-Li_2FeCl_4 and non-zero in o-Li_2FeCl_4, and associated with the emergence of superstructure reflections in the XRD patterns.

The structure factor F_{hkl} for this set of superstructure reflections is defined according to equation (3) where o_j is the occupancy, f_j is the atomic scattering factor, x_j, y_j, z_j are the atomic coordinates of the respective species, h, k, l are the Miller indices and N denotes the number of atoms in the motif:

$$F_{hkl} = \sum_{j=1}^{N} o_j \cdot f_j \cdot e^{-2\pi i(hx_j + ky_j + lz_j)} \tag{3}$$

The Debye-Waller factors were omitted from the structure factor for clarity. Since Li- and Fe(II)-ions occupy a site with the same atomic coordinates x_j, y_j, z_j, F_{hkl} will always contain a term that is linear in η:

$$F_{hkl} = \cdots + (o_{Fe}f_{Fe} + o_{Li}f_{Li})e^{-2\pi i(hx_j+ky_j+lz_j)} + \cdots \tag{4a}$$

$$= \cdots + (o_{Fe}f_{Fe} + o_{Li}f_{Li} + o_{Li}f_{Fe} - o_{Li}f_{Fe})e^{-2\pi i(hx_j+ky_j+lz_j)} + \cdots \tag{4b}$$

$$= \cdots + \big((\,o_{Fe} - o_{Li})f_{Fe} + o_{Li}(f_{Li} + f_{Fe})\big)e^{-2\pi i(hx_j+ky_j+lz_j)} + \cdots \tag{4c}$$

$$= \cdots + \left(\frac{o_{Fe}-o_{Li}}{o_{Fe}+o_{Li}} f_{Fe}(o_{Fe} + o_{Li}) + o_{Li}(f_{Li} + f_{Fe})\right) e^{-2\pi i(hx_j+ky_j+lz_j)} + \cdots \tag{4d}$$

$$= \cdots + \big(\eta f_{Fe}(o_{Fe} + o_{Li}) + o_{Li}(f_{Li} + f_{Fe})\big)e^{-2\pi i(hx_j+ky_j+lz_j)} + \cdots \tag{4f}$$

Hence, the superstructure reflections should increase linearly with temperature according to equation 5, in case of a second-order phase transition:

$$I_{hkl} \propto |F_{hkl}|^2 \propto \eta^2 \propto (T_c - T) \qquad (5)$$

3. Conductivity of $Li_{2-x}FeCl_4$

The ionic and electronic conductivity of o-Li_2FeCl_4 and o-$Li_{1.75}FeCl_4$ were determined by fitting the experimental impedance spectra to equivalent circuits. To ensure comparability between the different impedance spectra, the same, physically meaningful circuit model should be used for both samples. Since o-Li_2FeCl_4 is primarily an ionic conductor with negligible electronic conductivity, while o-$Li_{1.75}FeCl_4$ is a mixed ionic-electronic conductor (MIEC), the general circuit model must account for both edge cases. For a dense, conductive pellet without grain boundaries, a physically meaningful equivalent circuit can be derived by mapping the Nernst-Planck equation onto a general TML model featuring two parallel resistive rails with incremental resistances r_i ($i \in \mathrm{el}, \mathrm{ion}$) for ionic and electronic resistance respectively, coupled together by incremental chemical capacitors c_{chem}, as described by Jamnik and Maier,[8, 9] and further analyzed by Lai and Haile (Figure S4a).[10] The Jamnik-Maier TML has successfully been implemented in analyzing impedance spectra of LIBs before.[16, 17] Herein, the material properties are described by the incremental resistances r_i and capacitances c_{chem}, which add up to the total electronic and ionic resistance $R_i = \sum r_i$ and chemical capacitance $C_{\mathrm{chem}} = \sum c_{\mathrm{chem}}$ respectively, as well as the dielectric bulk capacitance C_{diel} of the conductive pellet, which is connected in parallel to the entire TML.[8-10, 18] For electronically conductive, but ionically blocking electrodes, like the stainless steel (SS) electrodes used herein, two additional circuit elements are introduced for the terminal impedances. The electronic and ionic rails are terminated by an electronic charge-transfer resistance $R_{\mathrm{ct,el}}$, and an interfacial capacitor $C_{\mathrm{el/ion}}$ respectively, given that side reactions at the electrodes can be neglected (Figure S4a).[10] The impedance of the TML is then given by equations 6, a to g, where Z is the impedance, i is the imaginary unit and ω is the angular frequency (rad s^{-1}):[8, 10]

$$Z_{\mathrm{TML}}(\omega) = Z_{\infty} + (Z_0 - Z_{\infty}) \cdot \frac{(1 + Z_{\mathrm{cross}}) \cdot \tanh(k)}{(k + Z_{\mathrm{cross}}) \cdot \tanh(k)} \tag{6a}$$

$$Z_{\infty} = \frac{Z_{\mathrm{ion}} \cdot Z_{\mathrm{el}}}{Z_{\mathrm{ion}} + Z_{\mathrm{el}}} + 2\frac{Z_{\mathrm{int,el}} \cdot Z_{\mathrm{int,ion}}}{Z_{\mathrm{int,el}} + Z_{\mathrm{int,ion}}} \tag{6b}$$

$$\frac{1}{Z_0} = \frac{1}{Z_{\mathrm{ion}} + 2Z_{\mathrm{ion}}} + \frac{1}{Z_{\mathrm{el}} + 2Z_{\mathrm{int,el}}} \tag{6c}$$

$$Z_{\mathrm{cross}} = \frac{Z_{\mathrm{ion}} + Z_{\mathrm{el}}}{2(Z_{\mathrm{int,el}} + Z_{\mathrm{int,ion}})} \tag{6d}$$

$$k = \frac{1}{2}\sqrt{\frac{Z_{\mathrm{ion}} \cdot Z_{\mathrm{el}}}{Z_{\mathrm{chem}}}} \tag{6e}$$

$$Z_{\mathrm{ion}} = R_{\mathrm{ion}} \qquad Z_{\mathrm{el}} = R_{\mathrm{el}} \qquad Z_{\mathrm{chem}} = \frac{1}{Q_{\mathrm{chem}}(i\omega)^{\alpha_{\mathrm{chem}}}} \tag{6f}$$

$$Z_{\mathrm{int,ion}} = \frac{1}{Q_{\mathrm{el/ion}}(i\omega)^{\alpha_{\mathrm{el/ion}}}} \qquad Z_{\mathrm{int,el}} = R_{\mathrm{ct,el}} \tag{6g}$$

Overall, the full TML is described by three resistors and three capacitors, leading to a maximum of three semicircles if the capacitances of the respective elements are sufficiently well resolved. The capacitors were modelled with CPEs ($C_i \rightarrow Q_i$) to account for non-ideal capacitors, and are subsequently denoted with Q_i.

The high-frequency semicircle arises from the $R_{\mathrm{bulk}}/Q_{\mathrm{diel}}$ element with the bulk resistance R_{bulk} of the conductor given by equation 7:[8, 18, 19]

$$R_{\mathrm{bulk}} = \frac{R_{\mathrm{ion}} \cdot R_{\mathrm{el}}}{R_{\mathrm{ion}} + R_{\mathrm{el}}} \tag{7}$$

Since R_{bulk} arises from R_{el} and R_{ion} connected in parallel, R_{bulk} typically reduces to the smaller of the two resistances and may therefore be interpreted as the ionic or electronic resistance if the conductor is predominantly ionically or electronically conductive.

The low-frequency feature on the other hand arises from the ionically blocking contact to the SS current collectors, and approaches the transport resistance of the electrons at low frequencies. For an ionic conductor with $R_{el} \rightarrow \infty$, the second semicircle becomes infinitely large and cannot be resolved, so the TML simplifies to the Debye circuit with R_{ion} in series with ½ $Q_{el/ion}$ and Q_{diel} in parallel (Figure S4b).[18, 19] Since no current flows though Q_{diel} at moderate to low frequencies, the Debye circuit is practically identical to a R_{ion}/Q_{diel} element in series ½ $Q_{el/ion}$ – a model that has been used in previous reports on ductile SSEs.[2, 20, 21]

For a MIEC, the low-frequency feature can be resolved. If $C_{chem} \ll C_{el/ion}$, the ionic and electronic rails approach a parallel circuit at low frequencies, and the low-frequency feature will appear as a second semicircle (Figure 4c).[8, 18] Meanwhile, the low-frequency feature simplifies to a half-teardrop shaped Warburg short (W_s) element for $C_{chem} \gg C_{el/ion}$, but only if $R_{ct,el} \rightarrow 0$ (Figure S4d).[8, 18] If $R_{ct,el}$ cannot be neglected, a third semicircle associated with the current collector interfaces may appear, and the TML can no longer be simplified.[8, 22]

o-Li_2FeCl_4 was fitted both with the TML, as well as the simplified Debye circuit (Table S9).[18, 19] The results from both fits were in very good agreement with each other. In line with the pellet acting purely as a dielectric in an ideal parallel plate capacitor at high frequencies, the respective CPE element had a CPE exponent α_{diel} close to 1. We note that the capacitance observed for the high-frequency semicircle C_{diel} was *ca.* 1 order of magnitude larger than expected from typical relative permittivity values in all samples ($Q_{diel} = 2 – 5 \cdot 10^{-10}$ F, TableS9). This is ascribed to stray capacitance from the impedance analyzer setup and was confirmed for a test R/C circuit with

well-defined resistance and capacitance. The ionic conductivity (0.0179(9) mS cm^{-1}) was in line with previous reports.[23, 24] The interfacial CPE coefficient $Q_{el/ion} = 10^{-6}$ F $s^{-0.15}$, as well as the slight non-ideality of the CPE (α=0.85) on the other hand, are in line with values typically expected for small space-charge layers along the uneven pellet in contact with the stainless steel current collector. Due to the very low electronic conductivity (*ca.* 10^{-6} mS cm^{-1}), only a small part of the low-frequency semicircle could be measured. The obtained conductivity therefore only indicates the order of magnitude, and an error of one order of magnitude was obtained (*vide infra*).

Since the measured impedance spectra of o-$Li_{1.75}FeCl_4$ contained all three semicircles, the full TML model was used for fitting (Table S9). o-$Li_{1.75}FeCl_4$ displayed much lower ionic and electronic resistance than o-Li_2FeCl_4. Within the TML, the resistance associated with the third semicircle at moderate frequencies is ascribed to $R_{ct,el}$ and therefore to the electronic rail. The correct origin of the semicircle is further suggested by the associated CPE coefficient $Q_{el/ion}$ = $4.4(4) \cdot 10^{-6}$ F $s^{-0.15}$, which is in line with typical values for interfacial capacitances, as well as the other measured samples. Nevertheless, it cannot be ruled out entirely that the semicircle may also be caused by any transverse interfacial impedance not considered in the Jamnik-Maier TML, e.g. due to the presence of grain boundaries,[19] or by inhomogeneous contact between the conductor and the stainless steel electrodes, leading to a breakdown of the approximation of a one-dimensional TML model.[25] Depending on the origin of the third semicircle, the associated resistance may have to be ascribed primarily to the ionic rather than the electronic rail, slightly decreasing R_{ion} and increasing R_{el}. Since the transverse resistance was small (108(3) Ω), the respective conductivities would not be altered significantly.

While the electronic conductivity of o-Li_2FeCl_4 and o-$Li_{1.75}FeCl_4$ was obtained from EIS fitting, the associated error for o-Li_2FeCl_4 was over one order of magnitude. A more commonly

employed method to measure electronic conductivity is DC polarization.[23, 24, 26, 27] Since the electronic conductivity of o-Li_2FeCl_4 is very low (*ca.* 10^{-5} – 10^{-6} mS cm^{-1}), the current response to an applied potential is very low, so higher potentials of >100 mV are often employed to improve the signal-to-noise ratio of the measured current.[23, 24] We noted in our experiments that o-Li_2FeCl_4 was not electrochemically stable at these potentials, as evidenced by a pronounced color change from white to black after prolonged DC polarization. Consistent with electrochemical decomposition, the DC current did not stabilize, even after hours of measurement. In order to obtain a reliable electrical resistance while preventing electrochemical decomposition, the DC potential needs to be as low as possible to follow Ohm's law. In order to determine a suitable potential, linear sweep voltammetry (LSV) was performed at very slow rates (2 μV s^{-1}) to simultaneously allow for the ionic motions to fully relax, thereby neglecting ionic conductivity, while also enabling the search for an appropriate potential for which Ohm's law was applicable (Figure S5, a to c). By fitting the LSV linearly according to Ohm's law for different potential windows (Figure S5c), it is clearly seen that the conductance is not constant, indicating a deviation from Ohm's law. In order to estimate the electronic conductance, the obtained conductances for different potential windows were extrapolated to 0 V. Because of the low signal-to-noise ratio in the current response of o-Li_2FeCl_4, only potential windows >30 mV were included for conductance extrapolation. We note that the DC current measures the total electronic resistance including $R_{ct,el}$, and the electronic conductivity is therefore slightly underestimated. For o-Li_2FeCl_4, the electronic resistance is dominated by the low electronic conductivity of the sample, and this effect can be neglected. For o-$Li_{1.75}FeCl_4$ with its higher electronic conductivity, $R_{ct,el}$, may however significantly increase the measured total resistance. The obtained electronic conductivities were 8.8(11) · 10^{-5} mS cm^{-1}, and 8.4(4) · 10^{-2} mS cm^{-1} for o-Li_2FeCl_4 and o-$Li_{1.75}FeCl_4$ respectively.

While these values are of the same order of magnitude like with those obtained from EIS fitting, the electronic conductivity of o-$Li_{1.75}FeCl_4$ obtained by LSV is slightly lower than from EIS fitting due to $R_{\mathrm{ct,el}}$.

Finally, the ambipolar conductivity $\tilde{\sigma}$, which describes the electroneutral combined motion of Li^+ and electrons, is defined by equation 8:

$$\tilde{\sigma} = \frac{\sigma_{\mathrm{ion}} \cdot \sigma_{\mathrm{el}}}{\sigma_{\mathrm{ion}} + \sigma_{\mathrm{el}}} \quad (8)$$

4. Phase Diagram of $Li_{2-x}FeCl4$

To map out the phase diagram of $Li_{2-x}FeCl4$, samples were mechanochemically synthesized from stoichiometric amounts of LiCl, $FeCl_2$ and $FeCl_3$ and subsequently thermally annealed (Methods). o-Li_2FeCl_4, o-$Li_{1.75}FeCl_4$ and $LiAlCl_4$-type Li_1FeCl_4 were initially synthesized and their XRD patterns refined (Figure S6, Table S10-S14). o-$Li_{1.75}FeCl_4$ crystallizes in the same cation-ordered spinel phase as o-Li_2FeCl_4, albeit slightly distorted, with similar *c*-axis, (Li_2FeCl_4: 10.32 Å, $Li_{1.75}FeCl_4$: 10.33 Å) but slightly contracted within the *ab* plane (Li_2FeCl_4: 7.31 Å, 7.31 Å, $Li_{1.75}FeCl_4$: 7.23 Å, 7.25 Å).

The degree of distortion can be quantified by relating the orthorhombic lattice parameters back to the cubic spinel unit cell, in which $a_c = b_c = c_c = c_o \approx \sqrt{2}\, b_o \approx \sqrt{2}\, a_o$. In o-$Li_2FeCl_4$, this relationship holds within <0.2% deviation, highlighting the pseudo-cubic symmetry, which is also evident from the fact that the XRD pattern almost retains the high symmetry of c-Li_2FeCl_4. Meanwhile, o-$Li_{1.75}FeCl_4$, is more strongly distorted with >1% deviation between the *ab* plane and the *c*-axis. As a result, the XRD pattern also shows significant peak splitting.

Intermediate compositions ($Li_{1.875}FeCl_4$ and $Li_{1.5}FeCl_4$) were composed of binary mixtures of the respective end members of the phase diagram, ruling out any additional phases within the phase diagram. The refined phases contained in the intermediate compositions had identical lattice parameters to the pure compounds, indicating negligible Li-ion solubility ranges for o-Li_2FeCl_4, o-$Li_{1.75}FeCl_4$ and $LiAlCl_4$-type Li_1FeCl_4. The respective fractions (e.g. 50 mol-% for o-Li_2FeCl_4 and o-$Li_{1.75}FeCl_4$) for $Li_{1.875}FeCl_4$ further confirmed that the phases are close to ideal stoichiometry.

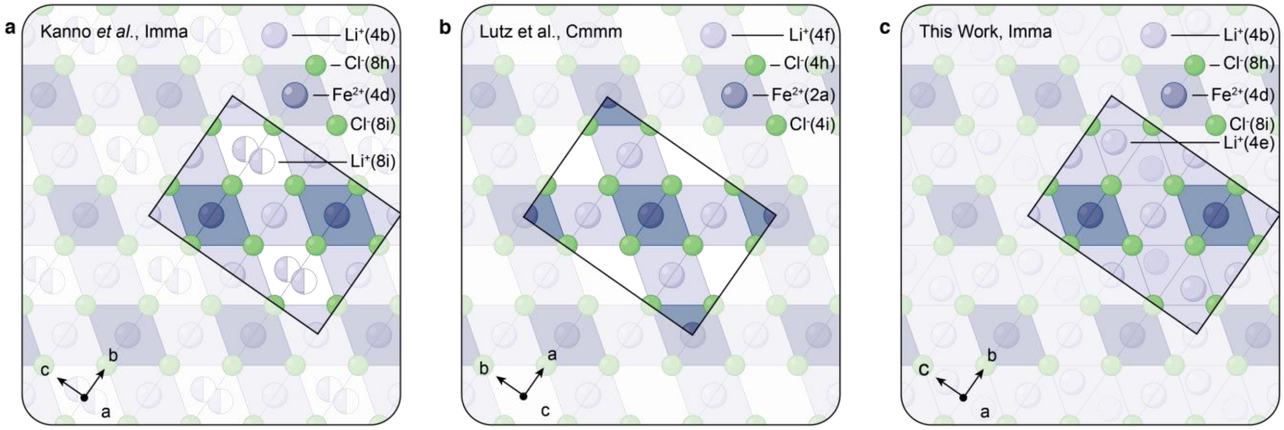

Figure S1. Schematic representation of the room-temperature structure of o-Li_2FeCl_4, with the *Imma* structure proposed by Kanno *et al.*[13] where Li-ions occupy a split position between tetrahedral and octahedral site (8i) (**a**), with the *Cmmm* structure proposed by Lutz *et al.*[28], where Li-ions occupy the octahedral site (4i) (**b**), and with the *Imma* structure proposed in this work, where Li-ions occupy the tetrahedral site (4e) of the cubic spinel aristotype (**c**).

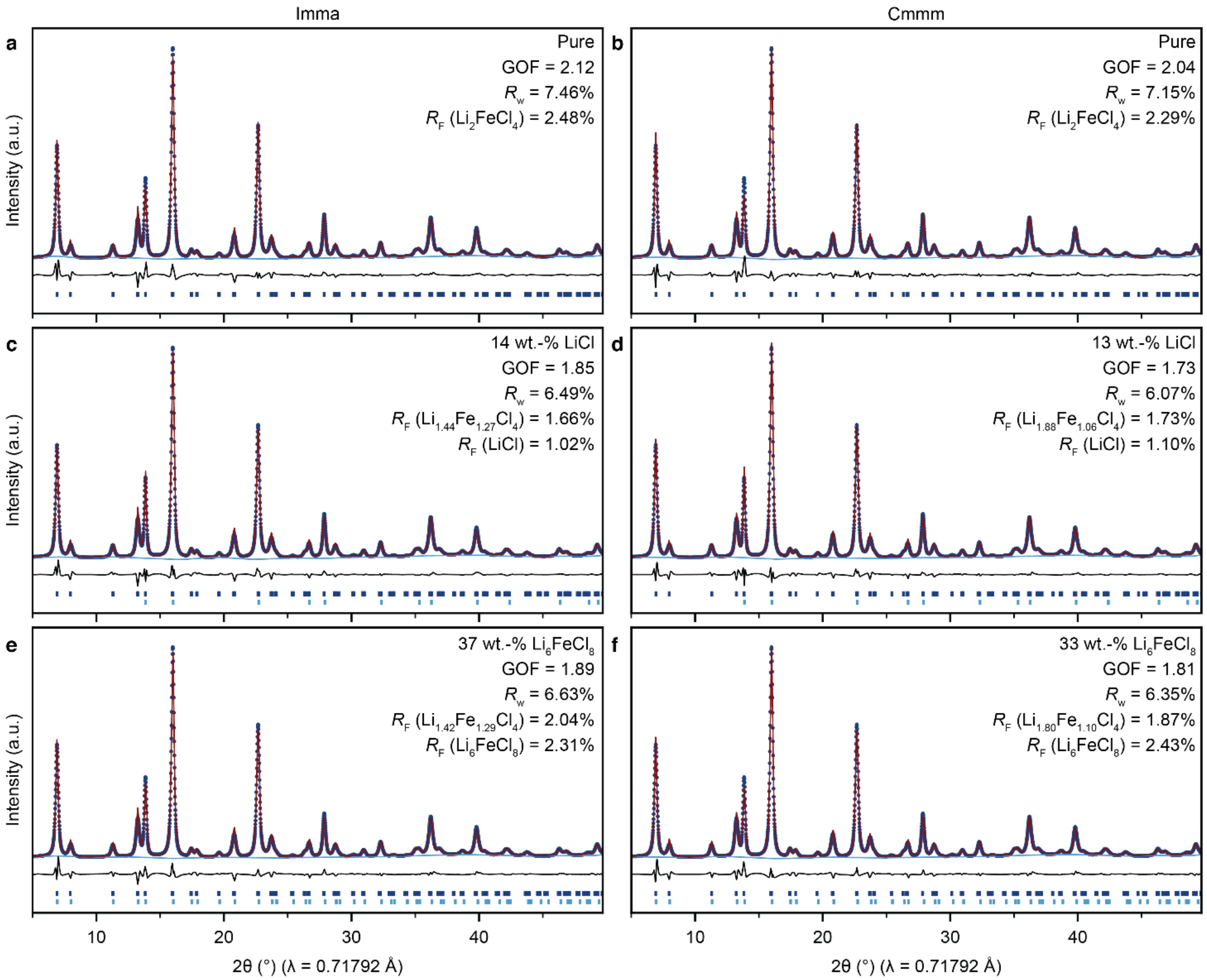

Figure S2. Rietveld refinement of the synchrotron powder diffraction patterns of as-synthesized o-Li_2FeCl_4. Refined in the space groups *Imma* (**a, c, e**) and *Cmmm* (**b, d, f**), without an additional impurity phase (**a, b**), with LiCl (**c, d**) and with Li_6FeCl_8 (**e, f**). Refinement results are given in Tables S1-S6.

Table S1. Overview of the Rietveld-refined parameters for as-synthesized o-Li_2FeCl_4 presented in Figure S2a. Values in brackets indicate estimated standard error. If no error is given, the parameter was not refined. Occupancies were constrained according to the stoichiometry (Li_2FeCl_4). U_{iso} was constrained for a given Wyckoff site.

S.G. *Imma*[13]	x = 100 wt.-%	d_{cryst} = 24.3(2) nm	ε_{micro} = 0.481(12)%
a = 7.3064(4) Å	b = 7.2908(5) Å	c = 10.3721(4) Å	$\alpha = \beta = \gamma = 90°$

Site	Wyck.	x	y	y	Occ.	U_{iso} (Å^2)
Li1	4e	0	¼	0.14	1	0.1
Li2	4b	0	0	½	0.842(1)	0.01
Fe1	4b	0	0	½	0.158(1)	0.01
Li3	4d	¼	¼	¾	0.158(1)	0.0157(3)
Fe2	4d	¼	¼	¾	0.842(1)	0.0157(3)
Cl1	8h	0	-0.0135(4)	0.2559(5)	1	0.0105(5)
Cl2	8i	0.2562(9)	¼	-0.0118(3)	1	0.0158(5)

N(Reflections) = 277	*N*(Obs.) = 17909	*N*(Params.) = 34	
χ^2 = 80621	GOF = 2.12	R_w = 7.46%	R_F = 2.48%

Table S2. Overview of the Rietveld-refined parameters for as-synthesized o-Li_2FeCl_4 presented in Figure S2b. Values in brackets indicate estimated standard error. If no error is given, the parameter was not refined. Occupancies were constrained according to the stoichiometry (Li_2FeCl_4). U_{iso} was constrained for a given Wyckoff site.

S.G. *Cmmm*[28]	*x* = 100 wt.-%	d_{cryst} = 23.6(1) nm	ε_{micro} = 0.386(11)%
a = 7.2930(4) Å	*b* = 10.3728(4) Å	*c* = 3.65231(17) Å	α = β = γ = 90°

Site	Wyck.	x	y	y	Occ.	U_{iso} (Å^2)
Li1	4f	¼	¼	½	0.953(1)	0.02
Fe1	4f	¼	¼	½	0.047(1)	0.02
Li2	2a	0	0	0	0.093(1)	0.0201(3)
Fe2	2a	0	0	0	0.907(1)	0.0201(3)
Cl1	4h	0.2382(2)	0	½	1	0.0095(3)
Cl2	4i	0	0.2368(2)	0	1	0.0129(3)

N(Reflections) = 160	*N*(Obs.) = 17909	*N*(Params.) = 33	
χ^2 = 74221	GOF = 2.04	R_w = 7.15%	R_F = 2.29%

Table S3. Overview of the Rietveld-refined parameters for as-synthesized o-Li_2FeCl_4 in the presence of LiCl presented in Figure S2c. Values in brackets indicate estimated standard error. If no error is given, the parameter was not refined. Occupancies were not constrained by stoichiometry because of the LiCl impurity. U_{iso} was constrained for a given Wyckoff site.

			Li_2FeCl_4			
S.G. *Imma*[13]		x = 85.57(19) wt.-%		d_{cryst} = 21.4(1) nm		ε_{micro} = 0.416(14)%
a = 7.3101(4) Å		b = 7.2925(5) Å		c = 10.3703(5) Å		$\alpha = \beta = \gamma = 90°$
Site	Wyck.	x	y	y	Occ.	U_{iso} (Å^2)
Li1	4e	0	¼	0.1959(13)	1	0.02
Li2	4b	0	0	½	0.470(4)	0.01
Fe1	4b	0	0	½	0.265(2)	0.01
Li3	4d	¼	¼	¾	0	0.0189(5)
Fe2	4d	¼	¼	¾	1	0.0189(5)
Cl1	8h	0	-0.0153(5)	0.2578(9)	1	0.0059(7)
Cl2	8i	0.2586(11)	¼	-0.0106(4)	1	0.0156(9)
N(Reflections) = 277				R_F = 1.66%		
			LiCl			
S.G. $Fm\bar{3}m$[29]		x = 14.43(19) wt.-%		d_{cryst} = 0.28(13) µm		ε_{micro} = 1.50(7)%
$a = b = c$ = 5.1611(4) Å				$\alpha = \beta = \gamma = 90°$		
Site	Wyck.	x	y	y	Occ.	U_{iso} (Å^2)
Li1	4a	0	0	0	1	0.01
Cl1	4b	½	½	½	1	0.0156(15)
N(Reflections) = 15				R_F = 1.02%		
N(Obs.) = 17909	N(Params.) = 51	χ^2 = 61141		GOF = 1.85		R_w = 6.49%

Table S4. Overview of the Rietveld-refined parameters for as-synthesized o-Li_2FeCl_4 in the presence of LiCl presented in Figure S2d. Values in brackets indicate estimated standard error. If no error is given, the parameter was not refined. Occupancies were not constrained by stoichiometry because of the LiCl impurity. U_{iso} was constrained for a given Wyckoff site.

			Li_2FeCl_4			
S.G. *Cmmm*[28]		x = 86.60(17) wt.-%		d_{cryst} = 21.5(1) nm		ε_{micro} = 0.373(11)%
a = 7.2926(4) Å		b = 10.3726(4) Å		c = 3.65390(16) Å		α = β = γ = 90°
Site	Wyck.	x	y	y	Occ.	U_{iso} (Å^2)
Li1	4f	¼	¼	½	0.874(2)	0.02
Fe1	4f	¼	¼	½	0.063(1)	0.02
Li2	2a	0	0	0	0	0.0129(3)
Fe2	2a	0	0	0	1	0.0129(3)
Cl1	4h	0.2348(2)	0	½	1	0.0097(3)
Cl2	4i	0	0.2377(2)	0	1	0.0141(5)
N(Reflections) = 160				R_F = 1.73%		
			LiCl			
S.G. $Fm\bar{3}m$[29]		x = 13.40(17) wt.-%		d_{cryst} = 0.13(6) µm		ε_{micro} = 1.18(8)%
$a = b = c$ = 5.1615(4) Å				α = β = γ = 90°		
Site	Wyck.	x	y	y	Occ.	U_{iso} (Å^2)
Li1	4a	0	0	0	1	0.01
Cl1	4b	½	½	½	1	0.0168(16)
N(Reflections) = 15				R_F = 1.10%		
N(Obs.) = 17909	N(Params.) = 38		χ^2 = 53373		GOF = 1.73	R_w = 6.07%

Table S5. Overview of the Rietveld-refined parameters for as-synthesized o-Li_2FeCl_4 in the presence of Li_6FeCl_8 presented in Figure S2e. Values in brackets indicate estimated standard error. If no error is given, the parameter was not refined. Occupancies were not constrained by stoichiometry because of the Li_6FeCl_8 impurity. U_{iso} was constrained for a given Wyckoff site.

			Li_2FeCl_4			
S.G. Imma[13]		x = 63.4(7) wt.-%		d_{cryst} = 20.3(2) nm		ε_{micro} = 0.13(2)%
a = 7.3129(5) Å		b = 7.2992(8) Å		c = 10.3798(6) Å		$\alpha = \beta = \gamma = 90°$
Site	Wyck.	x	y	y	Occ.	U_{iso} (Å^2)
Li1	4e	0	¼	0.14	1	0.1
Li2	4b	0	0	½	0.299(13)	0.01
Fe1	4b	0	0	½	0.351(6)	0.01
Li3	4d	¼	¼	¾	0.058(4)	0.0177(5)
Fe2	4d	¼	¼	¾	0.942(4)	0.0177(5)
Cl1	8h	0	-0.0130(12)	0.2554(8)	1	0.0059(10)
Cl2	8i	0.259(2)	¼	-0.0125(6)	1	0.0142(11)
N(Reflections) = 277				R_F = 2.04%		
			Li_6FeCl_8			
S.G. $Fm\bar{3}m$[14]		x = 36.6(7) wt.-%		d_{cryst} = 29.0(6) nm		ε_{micro} = 0.51(3)%
$a = b = c$ = 10.3113(5) Å				$\alpha = \beta = \gamma = 90°$		
Site	Wyck.	x	y	y	Occ.	U_{iso} (Å^2)
Li1	24d	0	¼	¼	1	0.038
Fe1	4a	0	0	0	1	0.01
Cl1	8c	¼	¼	¼	1	0.0214(14)
Cl2	24e	0.2399(4)	0	0	1	0.0214(14)
N(Reflections) = 71				R_F = 2.31%		
N(Obs.) = 17909		N(Params.) = 43	χ^2 = 63785	GOF = 1.89		R_w = 6.63%

Table S6. Overview of the Rietveld-refined parameters for as-synthesized o-Li_2FeCl_4 in the presence of Li_6FeCl_8 presented in Figure S2f. Values in brackets indicate estimated standard error. If no error is given, the parameter was not refined. Occupancies were not constrained by stoichiometry because of the Li_6FeCl_8 impurity. U_{iso} was constrained for a given Wyckoff site.

			Li_2FeCl_4			
S.G. *Cmmm*[28]		*x* = 68.3(4) wt.-%		d_{cryst} = 19.4(1) nm		ε_{micro} = 0.44(3)%
a = 7.3005(6) Å		*b* = 10.3779(5) Å		*c* = 3.65622(18) Å		α = β = γ = 90°
Site	Wyck.	x	y	y	Occ.	U_{iso} (Å^2)
Li1	4f	¼	¼	½	0.795(4)	0.02
Fe1	4f	¼	¼	½	0.103(2)	0.02
Li2	2a	0	0	0	0	0.0200(3)
Fe2	2a	0	0	0	1	0.0200(3)
Cl1	4h	0.2372(4)	0	½	1	0.0090(4)
Cl2	4i	0	0.2353(3)	0	1	0.0125(6)
N(Reflections) = 160				R_F = 1.87%		
			Li_6FeCl_8			
S.G. $Fm\bar{3}m$[14]		*x* = 32.7(4) wt.-%		d_{cryst} = 29.4(5) nm		ε_{micro} = 0.44(3)%
a = *b* = *c* = 10.3064(4) Å				α = β = γ = 90°		
Site	Wyck.	x	y	y	Occ.	U_{iso} (Å^2)
Li1	24d	0	¼	¼	1	0.038
Fe1	4a	0	0	0	1	0.01
Cl1	8c	¼	¼	¼	1	0.0212(12)
Cl2	24e	0.2405(4)	0	0	1	0.0149(6)
N(Reflections) = 71				R_F = 2.43%		
N(Obs.) = 17909	*N*(Params.) = 39		χ^2 = 58468	GOF = 1.81		R_w = 6.35%

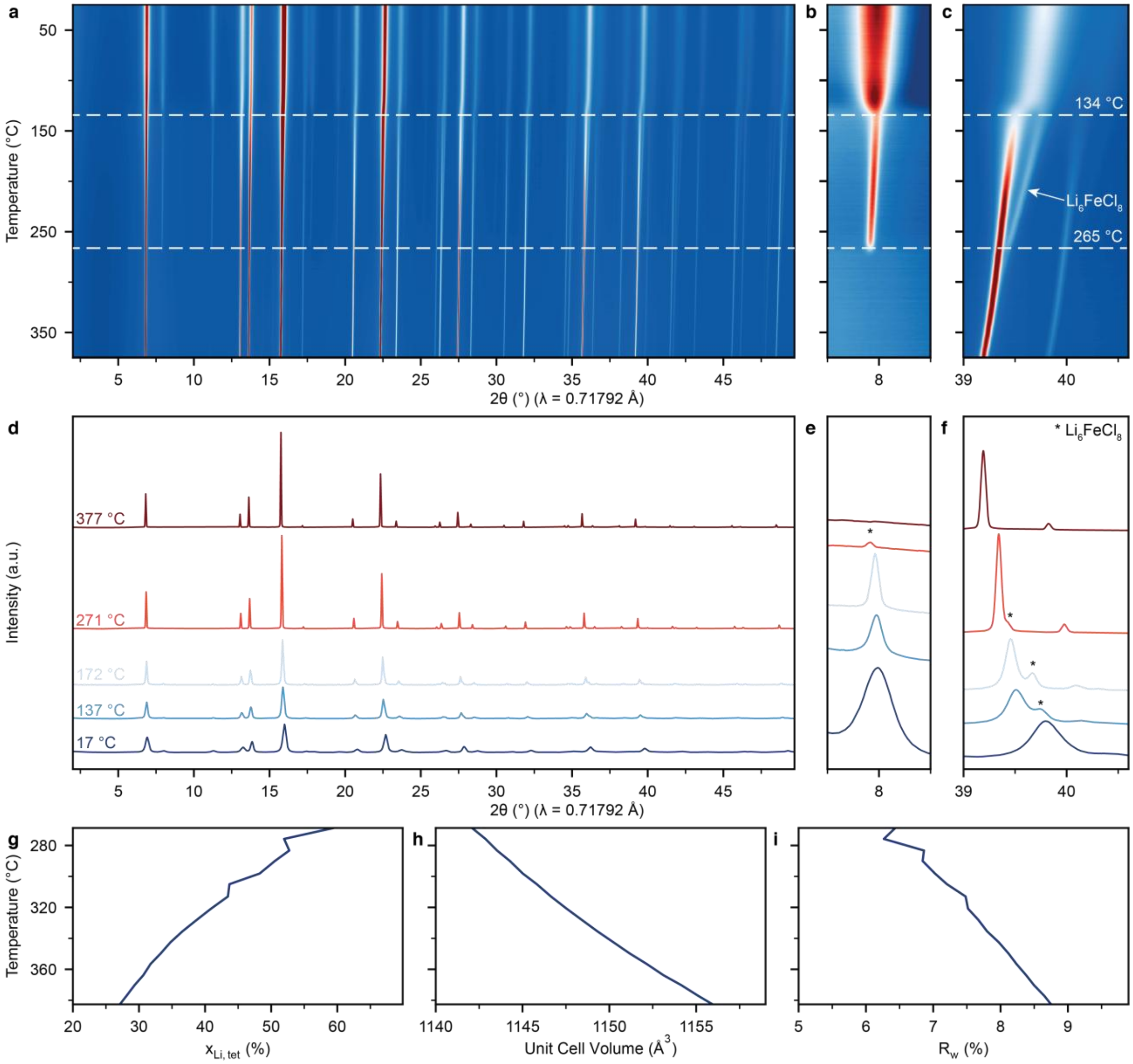

Figure S3. *In-situ* SXRD of as-prepared Li_2FeCl_4 during heating at 2 °C min^{-1} **(a-c)** and selected SXRD patterns **(d-f)**. Temperature evolution for selected Rietveld parameters of c-Li_2FeCl_4 during the *in-situ* SXRD (**g-i**). Fraction of tetrahedral Li relative to full occupation of the 8a site (**g**) and unit cell volume (**h**). The consistently low global residuals (R_w < 9%) (**i**) indicate the goodness of fit.

Table S7. Overview of the Rietveld-refined parameters for heat-treated o-Li_2FeCl_4 presented in Figure 2c. Values in brackets indicate estimated standard error. If no error is given, the parameter was not refined. Occupancies were constrained by stoichiometry. U_{iso} was constrained for a given Wyckoff site.

			Li_2FeCl_4			
S.G. *Imma*[13]		x = 98.93(4) wt.-%		d_{cryst} = 1.75(14) µm		ε_{micro} = 0.402(2)%
a = 7.31631(11) Å		b = 7.31654(12) Å		c = 10.33356(14) Å		α = β = γ = 90°
Site	Wyck.	x	y	y	Occ.	U_{iso} (Å^2)
Li1	4e	0	¼	0.0710(13)	1	0.05
Li2	4b	0	0	½	0.922(1)	0.01
Fe1	4b	0	0	½	0.078(1)	0.01
Li3	4d	¼	¼	¾	0.922(1)	0.01058(18)
Fe2	4d	¼	¼	¾	0.078(1)	0.01058(18)
Cl1	8h	0	-0.0184(3)	0.2484(12)	1	0.0126(3)
Cl2	8i	0.2574(10)	¼	-0.0091(3)	1	0.0184(5)
N(Reflections) = 275				R_F = 2.11%		
			LiCl			
S.G. $Fm\bar{3}m$[29]		x = 1.07(4) wt.-%		d_{cryst} = 10 µm		ε_{micro} = 0.01%
a = b = c = 5.1426(3) Å				α = β = γ = 90°		
Site	Wyck.	x	y	y	Occ.	U_{iso} (Å^2)
Li1	4a	0	0	0	1	0.01
Cl1	4b	½	½	½	1	0.014
N(Reflections) = 15				R_F = 6.54%		
N(Obs.) = 17909	N(Params.) = 55	χ^2 = 295545		GOF = 4.07		R_w = 8.48%

Table S8. Overview of the Rietveld-refined parameters for heat-treated c-Li_2FeCl_4 presented in Figure 2c. Values in brackets indicate estimated standard error. If no error is given, the parameter was not refined. Occupancies were not constrained by stoichiometry because of the LiCl impurity. U_{iso} was constrained for a given Wyckoff site.

Li_2FeCl_4						
S.G. Fd-3m[30]		x = 94.14(6) wt.-%		d_{cryst} = 1.07(3) µm		ε_{micro} = 0.1908(12)%
$a = b = c$ = 10.38014(3) Å				α = β = γ = 90°		
Site	Wyck.	x	y	y	Occ.	U_{iso} (Å^2)
Li1	8a	⅛	⅛	⅛	0.933(1)	0.0406(16)
Li2	16d	½	½	½	0.467(1)	0.01536(13)
Fe1	16d	½	½	½	0.533(1)	0.01536(13)
Cl1	32e	0.25710(2)	0.25710(2)	0.25710(2)	1	0.01597(10)
N(Reflections) = 65				R_F = 2.16%		
LiCl						
S.G. $Fm\bar{3}m$[29]		x = 5.86(6) wt.-%		d_{cryst} = 0.57(7) µm		ε_{micro} = 0.7%
$a = b = c$ = 5.14985(13) Å				α = β = γ = 90°		
Site	Wyck.	x	y	y	Occ.	U_{iso} (Å^2)
Li1	4a	0	0	0	1	0.01
Cl1	4b	½	½	½	1	0.080(5)
N(Reflections) = 15				R_F = 3.17%		
N(Obs.) = 19111		N(Params.) = 44	χ^2 = 142320	GOF = 2.73		R_w = 5.99%

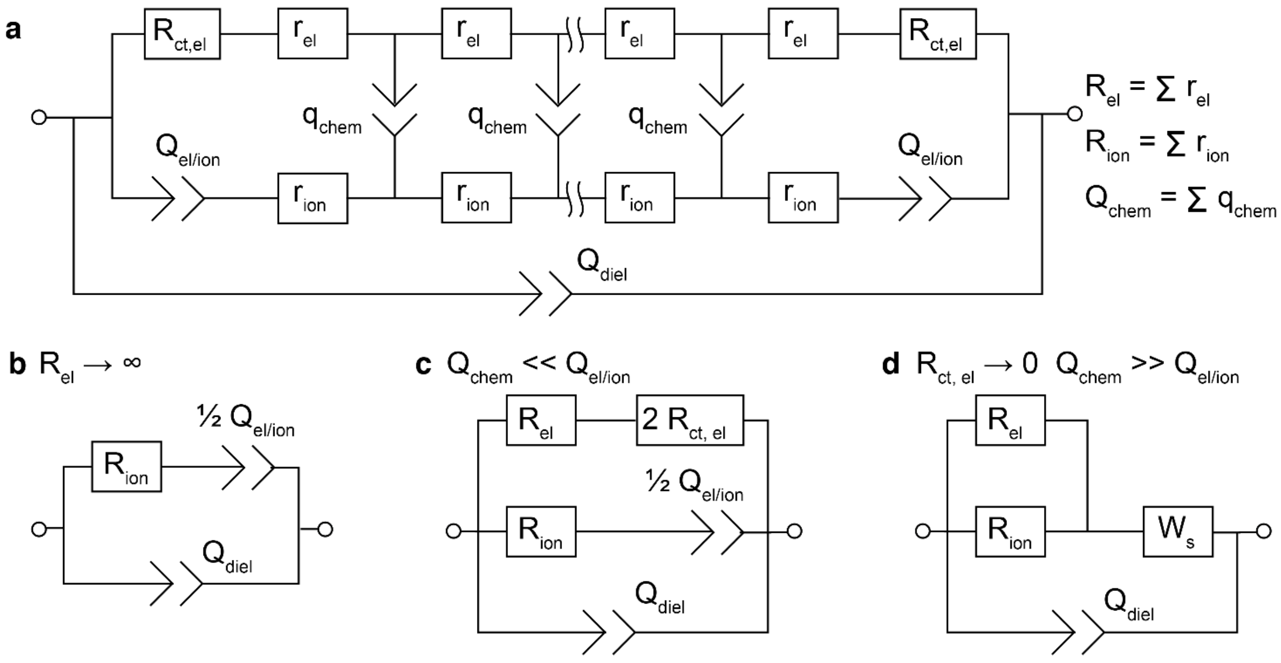

Figure S4. Equivalent circuits used to model the EIS data. General one-dimensional transmission line (TML) model for the transport of mass and charge across a MIEC slab (**a**). The circuit consists of two parallel resistive rails for electronic and ionic transport, coupled by chemical capacitors modelled with CPEs. The terminal impedances with the electronically conductive, but ionically insulating electrodes are modelled by a charge-transfer resistance for the electronic rail and a CPE for the ionic rail. The bulk dielectric capacitance of the MIEC is connected in parallel to the TML. (**b-d**) Equivalent circuits obtained from the general TML model for a SSE slab (**b**) and a MIEC slab with different relative CPE coefficients for Q_{chem} and $Q_{el/ion}$ (**c, d**). Fitted values for the different materials are summarized in Table S9.

Table S9. Overview of the refined parameters from fitting the experimental impedance data measured at 30 °C (Figure 3, c to e) to the respective equivalent circuits presented in Figure S4.

Sample	Li_2FeCl_4		$Li_{1.75}FeCl_4$
d (cm)	$6.1() \cdot 10^{-2}$		$6.9(2) \cdot 10^{-2}$
A (cm^2)	$4.6(2) \cdot 10^{-1}$		$4.6(2) \cdot 10^{-1}$
Model	Figure S4a	Figure S4b	Figure S4a
R_{ion} (Ω)	$7.05(8) \cdot 10^{3}$	$6.88(9) \cdot 10^{3}$	$3.622(18) \cdot 10^{2}$
σ_{ion} (mS cm^{-1})	$1.79(9) \cdot 10^{-2}$	$1.87(10) \cdot 10^{-2}$	$3.95(18) \cdot 10^{-1}$
Q_{diel} (F $s^{\alpha_{diel}-1}$)	$6.4(19) \cdot 10^{-10}$	$5(4) \cdot 10^{-10}$	$5.1(3) \cdot 10^{-8}$
α_{diel}	0.962(9)	1	0.696(3)
$Q_{el,ion}$ (F $s^{\alpha_{el,ion}-1}$)	$1.9(9) \cdot 10^{-6}$	$2.18(4) \cdot 10^{-6}$	$4.4(4) \cdot 10^{-6}$
$\alpha_{el,ion}$	0.85(8)	0.830(5)	0.750(9)
R_{el} (Ω)	$1(40) \cdot 10^{8}$		$1.380(11) \cdot 10^{3}$
σ_{el} (mS cm^{-1})	$1(40) \cdot 10^{-6}$		$1.04(5) \cdot 10^{-1}$
$R_{ct,el}$ (Ω)	$1(60000) \cdot 10^{2}$		$1.08(3) \cdot 10^{2}$
Q_{chem} (F $s^{\alpha_{chem}-1}$)	$2.1(7) \cdot 10^{-5}$		$2.35(3) \cdot 10^{2}$
α_{chem}	0.94(18)		0.595(5)

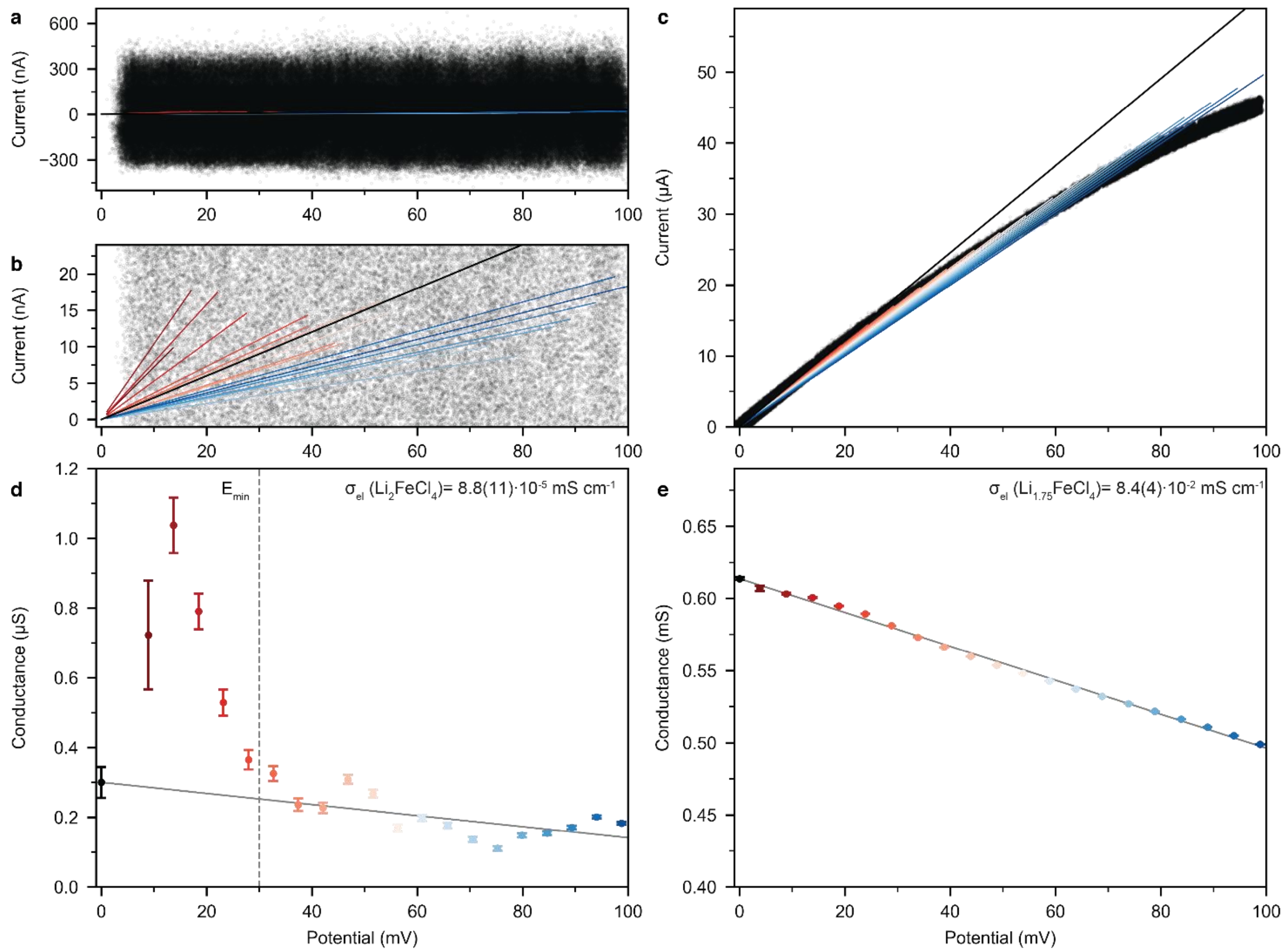

Figure S5. Linear Sweep Voltammetry (LSV) of Li_2FeCl_4, (**a, b**) and $Li_{1.75}FeCl_4$ (**c**). Since the LSV did not follow Ohm's law due to significant electrochemical side reactions, the LSV was extrapolated according to Ohm's law for different potential windows (colored lines), and the corresponding conductances (**d, e**) were extrapolated to 0 V to get an upper bound for the real conductance. Because of the high signal-to-noise ratio for Li_2FeCl_4, only potential windows >30 mV were included for conductance extrapolation.

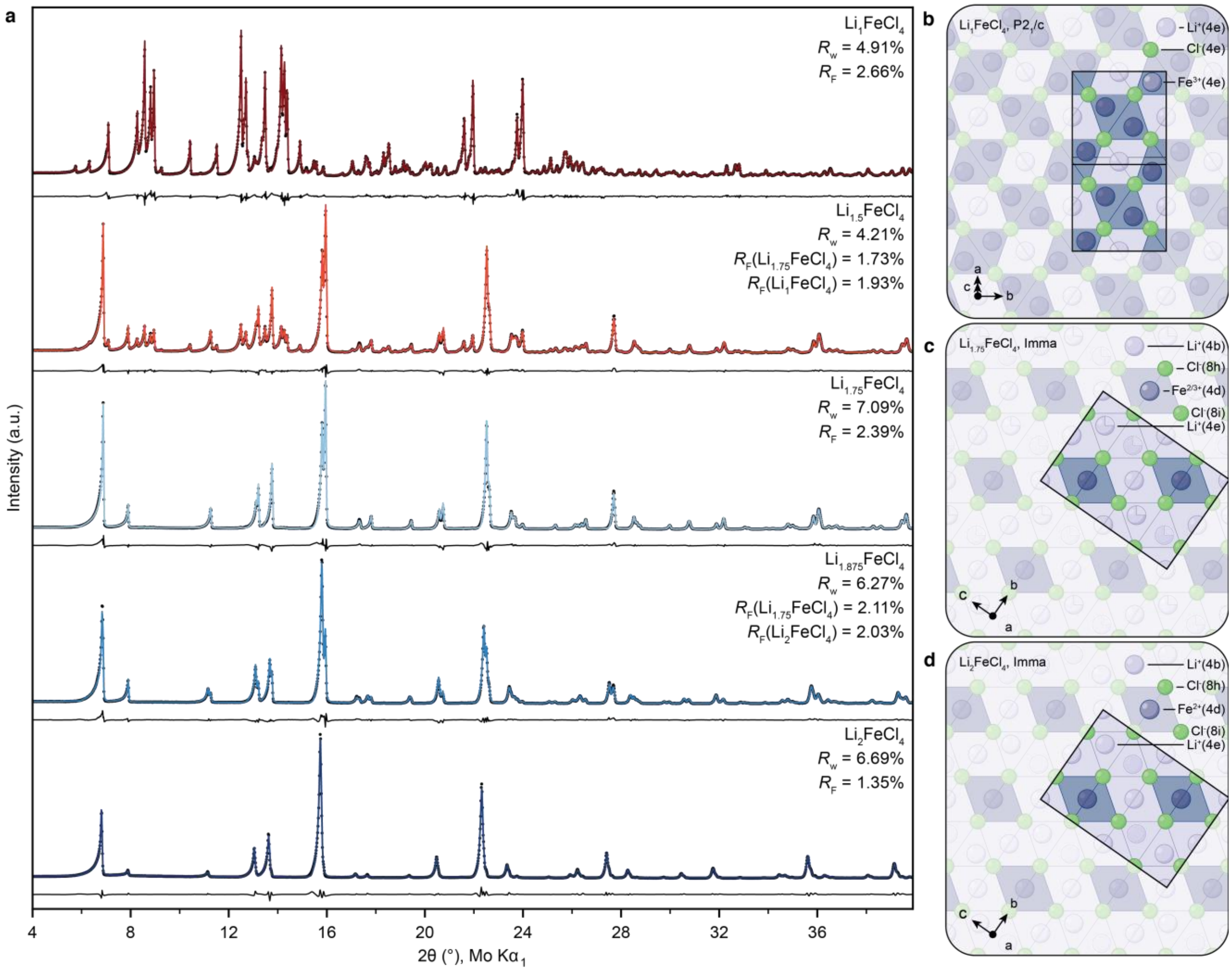

Figure S6. Rietveld refinements for synthesized $Li_{2-x}FeCl_4$ (0<x<1) samples at different lithium contents (**a**) and schematic representation of the structures of o-Li_2FeCl_4, (*Imma*) (**b**), o-$Li_{1.75}FeCl_4$ (*Imma*) (**c**) and Li_1FeCl_4 (*P2₁/c*) (**d**). Refined values can be found in Tables S10-S14.

Table S10. Overview of the Rietveld-refined parameters for heat-treated Li_2FeCl_4 in the presence of Li_6FeCl_8 presented in Figure 3b and S6a. Values in brackets indicate estimated standard error. If no error is given, the parameter was not refined. Occupancies were not constrained by stoichiometry because of the Li_6FeCl_8 impurity. U_{iso} was constrained for a given Wyckoff site.

			Li_2FeCl_4			
S.G. *Imma*[13]		x = 72.4(10) wt.-%		d_{cryst} = 1 µm		ε_{micro} = 0.415(10)%
a = 7.3396(9) Å		b = 7.3361(9) Å		c = 10.3632(7) Å		$\alpha = \beta = \gamma = 90°$
Site	Wyck.	x	y	y	Occ.	U_{iso} (Å^2)
Li1	4e	0	¼	0.108(13)	1	0.05
Li2	4b	0	0	½	0.06(3)	0.01
Fe1	4b	0	0	½	0.471(12)	0.01
Li3	4d	¼	¼	¾	0.249(8)	0.0243(14)
Fe2	4d	¼	¼	¾	0.751(8)	0.0243(14)
Cl1	8h	0	-0.020(2)	0.2563(17)	1	0.0060(19)
Cl2	8i	0.255(3)	¼	-0.0089(15)	1	0.018(2)
N(Reflections) = 161				R_F = 1.35%		
			Li_6FeCl_8			
S.G. $Fm\bar{3}m$[14]		x = 27.7(10) wt.-%		d_{cryst} = 1 µm		ε_{micro} = 0.562(19)%
$a = b = c$ = 10.3369(7) Å				$\alpha = \beta = \gamma = 90°$		
Site	Wyck.	x	y	y	Occ.	U_{iso} (Å^2)
Li1	24d	0	¼	¼	1	0.038
Fe1	4a	0	0	0	1	0.01
Cl1	8c	¼	¼	¼	1	0.0396(17)
Cl2	24e	0.2399	0	0	1	0.0396(17)
N(Reflections) = 44				R_F = 2.90%		
N(Obs.) = 2401	N(Params.) = 40	χ^2 = 129809		GOF = 7.41		R_w = 6.69%

Table S11. Overview of the Rietveld-refined parameters for heat-treated $Li_{1.875}FeCl_4$ presented in Figure S6a. Values in brackets indicate estimated standard error. If no error is given, the parameter was not refined. Occupancies were constrained by stoichiometry. U_{iso} was constrained for a given element within each phase.

			Li_2FeCl_4			
S.G. *Imma*[13]		x = 51.6(4) wt.-%		d_{cryst} = 1 µm		ε_{micro} = 0.357(8)%
a = 7.3124(6) Å		b = 7.3129(6) Å		c = 10.3219(5) Å		$\alpha = \beta = \gamma = 90°$
Site	Wyck.	x	y	y	Occ.	U_{iso} (Å^2)
Li1	4e	0	¼	0.125	1	0.05
Li2	4b	0	0	½	0.948(4)	0.03
Fe1	4b	0	0	½	0.052(4)	0.03
Li3	4d	¼	¼	¾	0.052(4)	0.0143(8)
Fe2	4d	¼	¼	¾	0.948(4)	0.0143(8)
Cl1	8h	0	-0.0181(8)	0.249(2)	1	0.0189(7)
Cl2	8i	0.2623(16)	¼	-0.0117(8)	1	0.0189(7)
N(Reflections) = 159				R_F = 2.03%		
			$Li_{1.75}FeCl_4$			
S.G. *Imma*[13]		x = 48.4(4) wt.-%		d_{cryst} = 1 µm		ε_{micro} = 0.284(6)%
a = 7.2365(2) Å		b = 7.2523(3) Å		c = 10.3305(4) Å		$\alpha = \beta = \gamma = 90°$
Site	Wyck.	x	y	y	Occ.	U_{iso} (Å^2)
Li1	4e	0	¼	0.125	¾	0.05
Li2	4b	0	0	½	0.958(4)	0.03
Fe1	4b	0	0	½	0.042(4)	0.03
Li3	4d	¼	¼	¾	0.042(4)	0.0162(9)
Fe2	4d	¼	¼	¾	0.958(4)	0.0162(9)
Cl1	8h	0	-0.0152(5)	0.2543(19)	1	0.0224(8)
Cl2	8i	0.252(3)	¼	-0.0139(5)	1	0.0224(8)
N(Reflections) = 149				R_F = 2.11%		
N(Obs.) = 2401	N(Params.) = 45		χ^2 = 23989	GOF = 3.19		R_w = 6.27%

Table S12. Overview of the Rietveld-refined parameters for heat-treated $Li_{1.75}FeCl_4$ presented in Figure 3b and S6a. Values in brackets indicate estimated standard error. If no error is given, the parameter was not refined. Occupancies were constrained by stoichiometry. U_{iso} was constrained for a given element within each phase.

			$Li_{1.75}FeCl_4$			
S.G. *Imma*[13]		x = 98.26(12) wt.-%		d_{cryst} = 1 µm		ε_{micro} = 0.251(3)%
a = 7.23345(16) Å		b = 7.2464(2) Å		c = 10.3305(2) Å		$\alpha = \beta = \gamma = 90°$
Site	Wyck.	x	y	y	Occ.	U_{iso} (Å^2)
Li1	4e	0	¼	0.125	¾	0.05
Li2	4b	0	0	½	0.942(2)	0.03
Fe1	4b	0	0	½	0.058(2)	0.03
Li3	4d	¼	¼	¾	0.058(2)	0.0159(5)
Fe2	4d	¼	¼	¾	0.942(2)	0.0159(5)
Cl1	8h	0	-0.0156(3)	0.2527(14)	1	0.0215(5)
Cl2	8i	0.2552(16)	¼	-0.0144(3)	1	0.0215(5)
N(Reflections) = 149				R_F = 2.39%		
			Li_1FeCl_4			
S.G. *P2₁/c*[31]		x = 1.74(12) wt.-%		d_{cryst} = 1 µm		ε_{micro} = 0.3%
a = 7.104(5) Å	b = 6.424(3) Å		c = 12.963(12) Å	$\alpha = \gamma = 90°$		β = 93.58(6)%
Site	Wyck.	x	y	y	Occ.	U_{iso} (Å^2)
Li1	4e	0.15688	0.98308	0.36658	1	0.03
Fe1	4e	0.70400	0.32170	0.89895	1	0.027
Cl1	4e	0.69220	0.18270	0.05079	1	0.04
Cl2	4e	0.81250	0.63560	0.93220	1	0.04
Cl3	4e	0.92720	0.17850	0.81360	1	0.04
Cl4	4e	0.43550	0.31550	0.81114	1	0.04
N(Reflections) = 551				R_F = 7.33%		
N(Obs.) = 2401	N(Params.) = 38		χ^2 = 143177	GOF = 7.78		R_w = 7.09%

Table S13. Overview of the Rietveld-refined parameters for heat-treated $Li_{1.5}FeCl_4$ presented in Figure S6a. Values in brackets indicate estimated standard error. If no error is given, the parameter was not refined. Occupancies were constrained by stoichiometry. U_{iso} was constrained for a given element within each phase.

			$Li_{1.75}FeCl_4$			
S.G. *Imma*[13]		x = 66.89(19) wt.-%		d_{cryst} = 1 µm		ε_{micro} = 0.328(3)%
a = 7.23035(15) Å		b = 7.24295(18) Å		c = 10.32621(18) Å		α = β = γ = 90°
Site	Wyck.	x	y	y	Occ.	U_{iso} (Å^2)
Li1	4e	0	¼	0.125	¾	0.05
Li2	4b	0	0	½	0.950(2)	0.04(4)
Fe1	4b	0	0	½	0.050(2)	0.04(4)
Li3	4d	¼	¼	¾	0.050(2)	0.0183(5)
Fe2	4d	¼	¼	¾	0.950(2)	0.0183(5)
Cl1	8h	0	-0.0155(3)	0.2542(10)	1	0.0208(4)
Cl2	8i	0.2550(15)	¼	-0.0150(2)	1	0.0208(4)
N(Reflections) = 149				R_F = 1.73%		
			Li_1FeCl_4			
S.G. *P2₁/c*[31]		x = 33.11(19) wt.-%		d_{cryst} = 1 µm		ε_{micro} = 0.303(7)%
a = 7.1003(3) Å	b = 6.4226(2) Å	c = 12.9449(7) Å		α = γ = 90°		β = 93.645(3)%
Site	Wyck.	x	y	y	Occ.	U_{iso} (Å^2)
Li1	4e	0.15688	0.98308	0.36658	1	0.03
Fe1	4e	0.7035(6)	0.3198(6)	0.8991(4)	1	0.0262(14)
Cl1	4e	0.6936(9)	0.1755(11)	0.0513(6)	1	0.0445(13)
Cl2	4e	0.8146(10)	0.6415(11)	0.9332(6)	1	0.0445(13)
Cl3	4e	0.9300(11)	0.1869(14)	0.8135(7)	1	0.0445(13)
Cl4	4e	0.4333(10)	0.3207(12)	0.8093(6)	1	0.0445(13)
N(Reflections) = 550				R_F = 1.93%		
N(Obs.) = 2401	N(Params.) = 57	χ^2 = 51460		GOF = 4.69		R_w = 4.21%

Table S14. Overview of the Rietveld-refined parameters for heat-treated Li_1FeCl_4 presented in Figure 3b and S6a. Values in brackets indicate estimated standard error. If no error is given, the parameter was not refined. U_{iso} was constrained for a given element.

			Li_1FeCl_4			
S.G. $P2_1/c$[31]		x = 100 wt.-%		d_{cryst} = 1 µm		ε_{micro} = 0.171(3)%
a=7.09988(14) Å	b=6.42167(12) Å		c=12.9411(3) Å	$\alpha = \gamma = 90°$		β = 93.6467(13)%
Site	Wyck.	x	y	y	Occ.	U_{iso} (Å^2)
Li1	4e	0.15688	0.98308	0.36658	1	0.02
Fe1	4e	0.7041(3)	0.3218(3)	0.89883(16)	1	0.0283(6)
Cl1	4e	0.6923(4)	0.1822(5)	0.0507(3)	1	0.0405(6)
Cl2	4e	0.8125(4)	0.6364(5)	0.9322(3)	1	0.0405(6)
Cl3	4e	0.9284(5)	0.1785(6)	0.8135(3)	1	0.0405(6)
Cl4	4e	0.4356(4)	0.3158(6)	0.8110(3)	1	0.0405(6)
N(Reflections) = 550				R_F = 2.66%		
N(Obs.) = 2467	N(Params.) = 44		χ^2 = 75938	GOF = 5.60		R_w = 4.91%

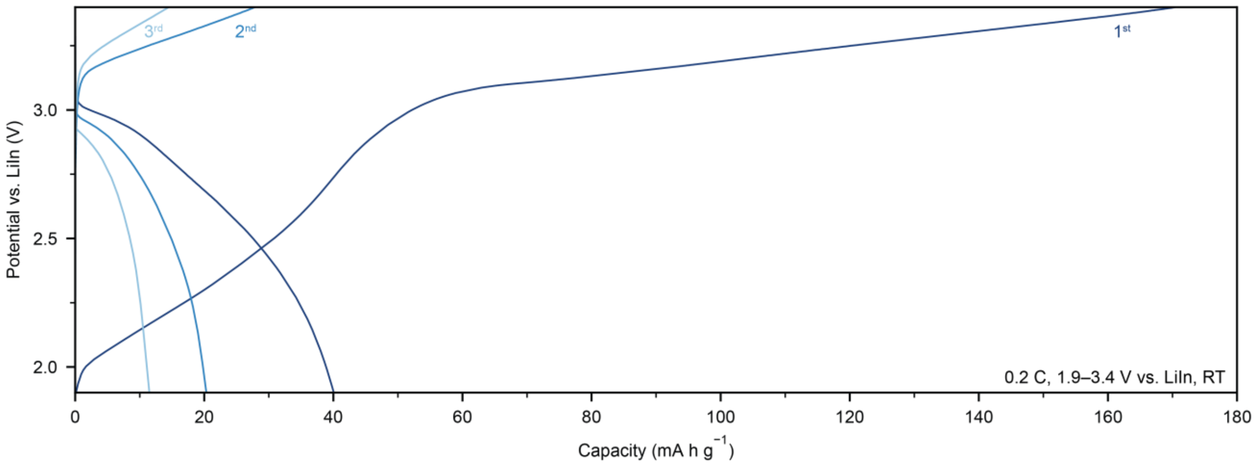

Figure S7. Voltage profiles for the first three cycles of all-solid-state batteries with cathodes containing Li_2FeCl_4 and Li_6PS_5Cl SSE.

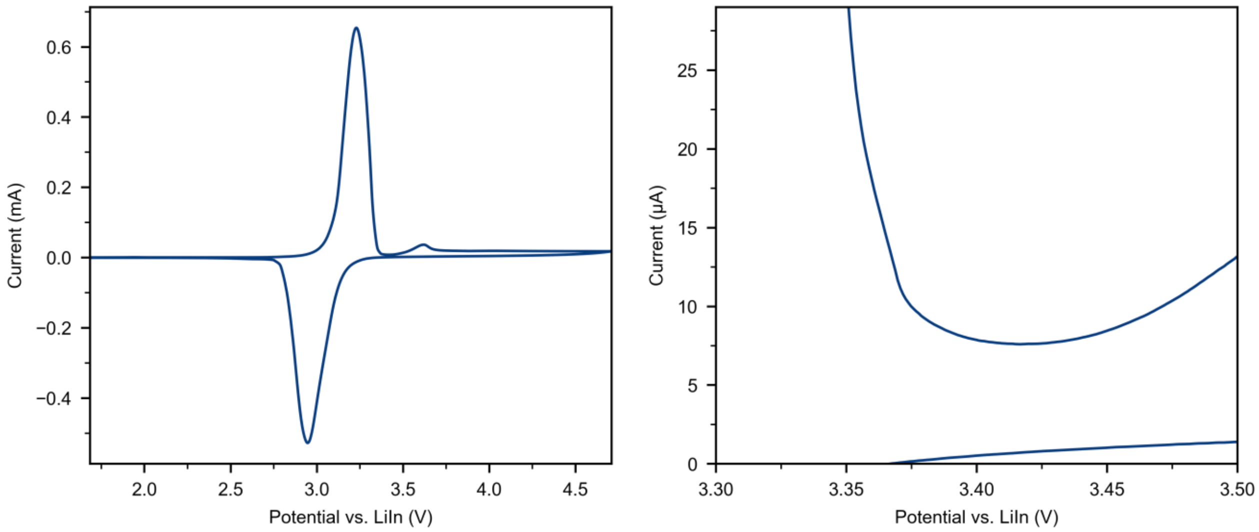

Figure S8. (a, b) Cyclic Voltammetry of all-solid-state batteries with cathodes containing Li_2FeCl_4 at 0.2 mV s^{-1}.

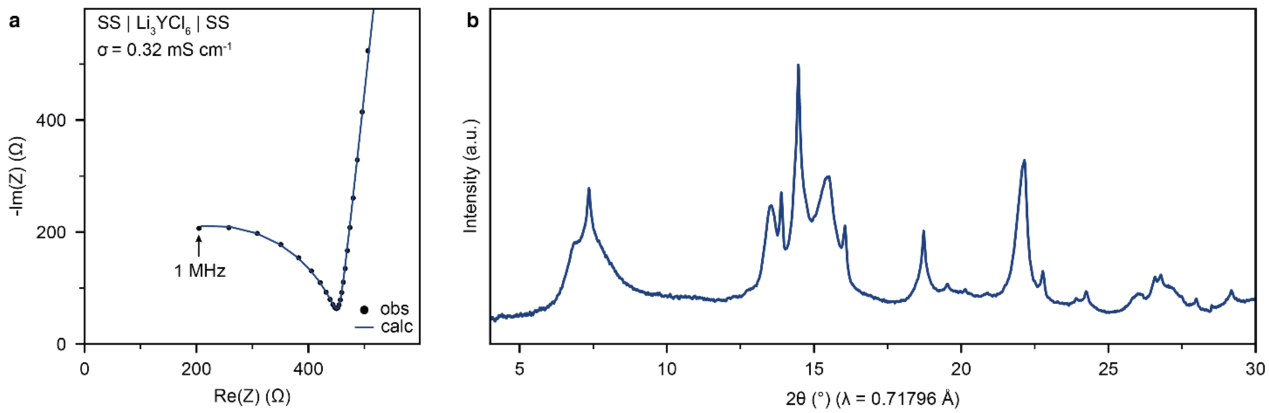

Figure S9. (**a**) Nyquist diagram of the ionic conductivity at room temperature of Li_3YCl_6 (LYC). (**b**) SXRD pattern of LYC.

Table S9. Overview of the refined parameters for Li_3YCl_6 presented in Figure S8 from fitting the experimental impedance data measured at 30 °C to the respective equivalent circuits presented in Figure S4.

Sample	Li_3YCl_6
d (cm)	$7.3(2) \cdot 10^{-2}$
A (cm^2)	$5.0(2) \cdot 10^{-1}$
Model	Figure S4b
R_{ion} (Ω)	$4.520(13) \cdot 10^{2}$
σ_{ion} (mS cm^{-1})	$3.23(16) \cdot 10^{-1}$
Q_{diel} (F $s^{\alpha_{diel}-1}$)	$8.3(5) \cdot 10^{-10}$
α_{diel}	0.9506(3)
Q_{elion} (F $s^{\alpha_{el,ion}-1}$)	$4.24(13) \cdot 10^{-7}$
$\alpha_{el,ion}$	0.931(3)

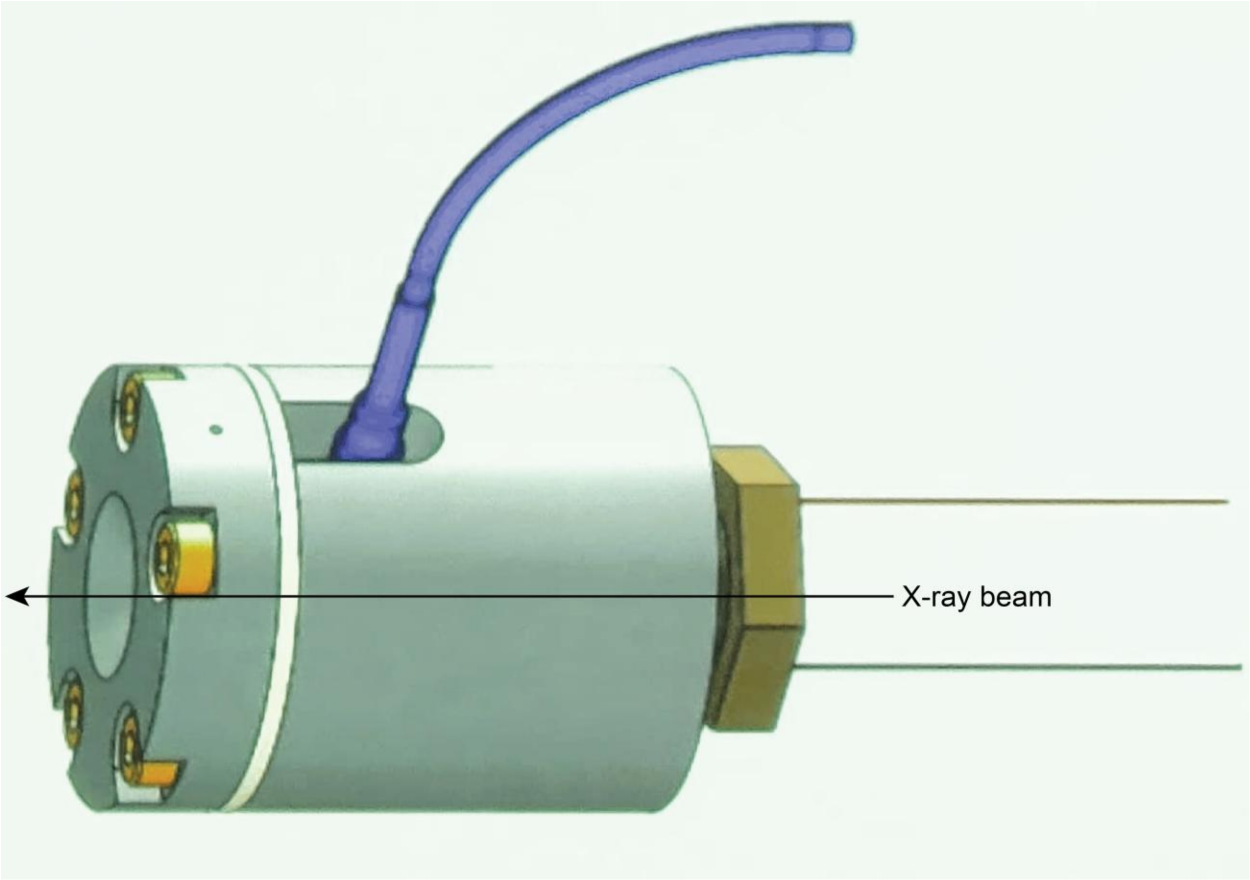

Figure S10. Operando SXRD cell setup.

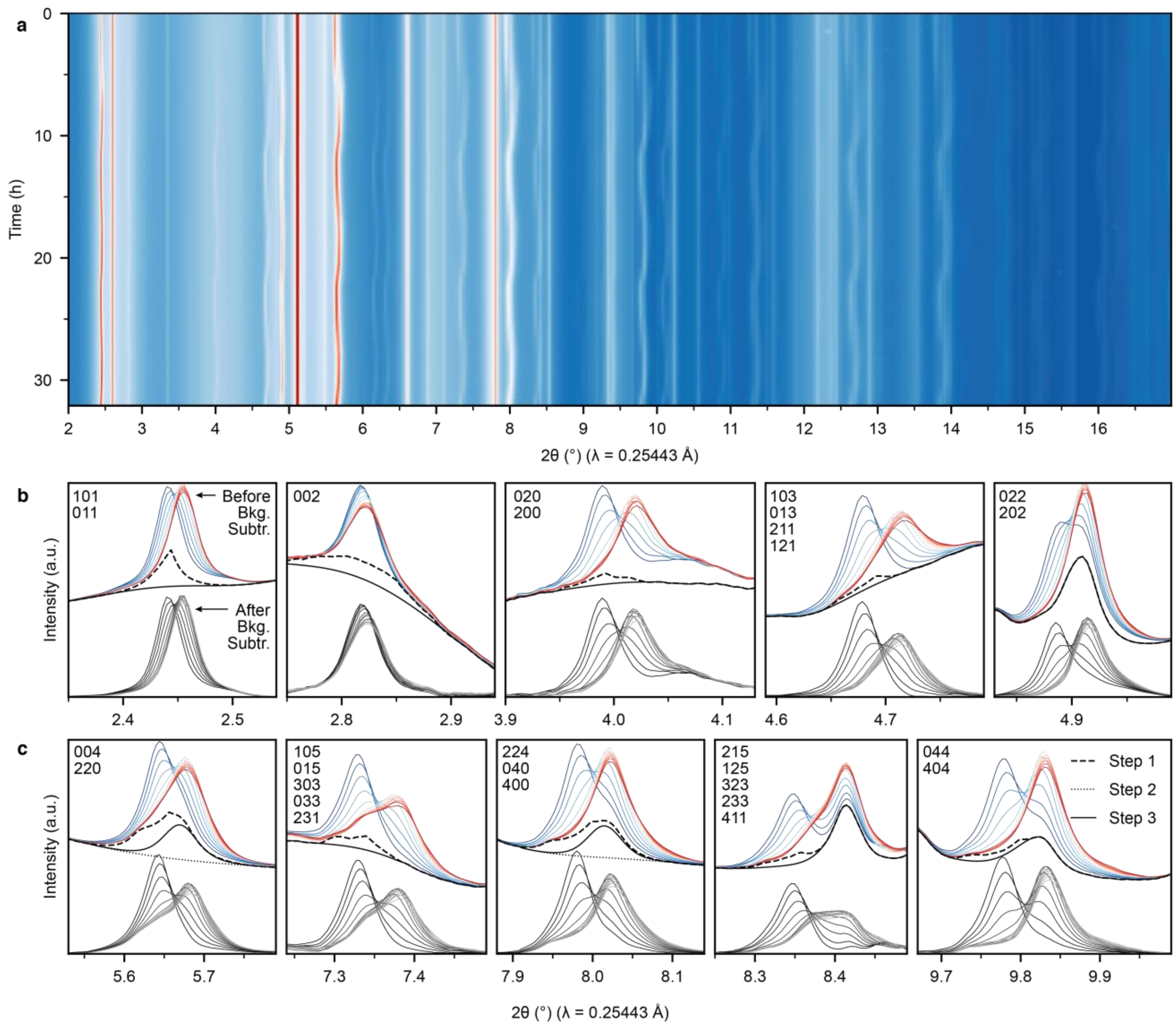

Figure S11. (**a**) *In-situ* SXRD of all-solid-state batteries with cathodes containing Li_2FeCl_4 before background subtraction. A logarithmic intensity scale was used to enhance low-intensity reflections. (**b, c**) Background determination highlighted for selected LFC reflections during the 1st cycle (blue/red lines). Indexing was done in the *Imma* space group. The background was determined by differentiating the SXRD with respect to time and reintegrating ($\int \frac{\partial I(2\theta,t)}{\partial t} dt + C(2\theta)$ with $C(2\theta) \geq 0$, Step 1, dashed line), performing a polynomial fit around peaks where differentiation/reintegration partially subtracted Li_2FeCl_4 intensity (Step 2, dotted line), and subtracting overlapping reflections of the LYC SSE (Step 3, black line). Grey lines indicate the LFC reflections after background subtraction.

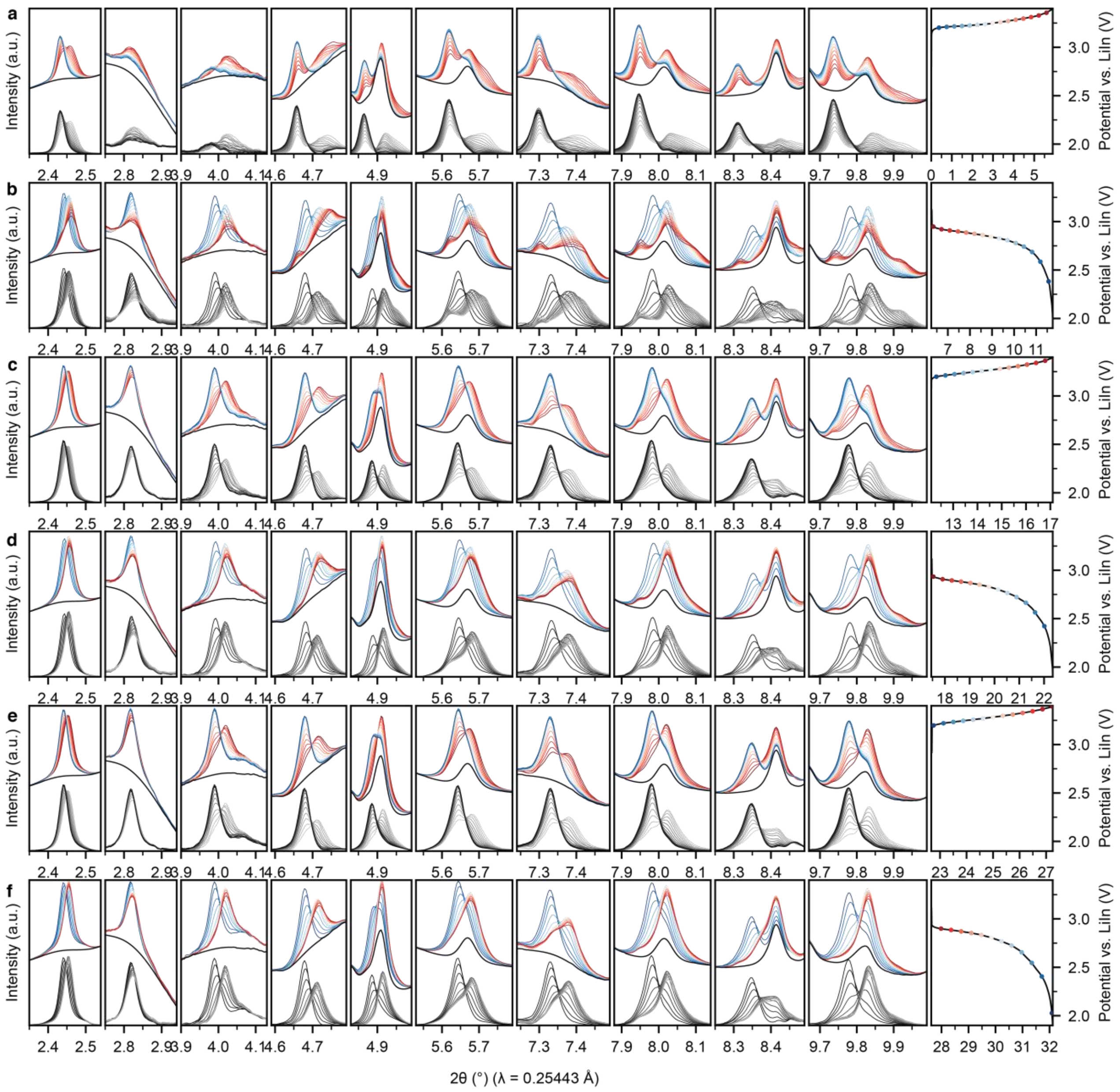

Figure S12. (**a-f**) *In-situ* SXRD of all-solid-state batteries with cathodes containing Li_2FeCl_4 before (blue/red lines) and after background subtraction (grey lines) with the background highlighted in black. On the right, voltage profile of the all-solid-state battery (black line) are plotted with the points of SXRD acquisition highlighted (blue/red dots).

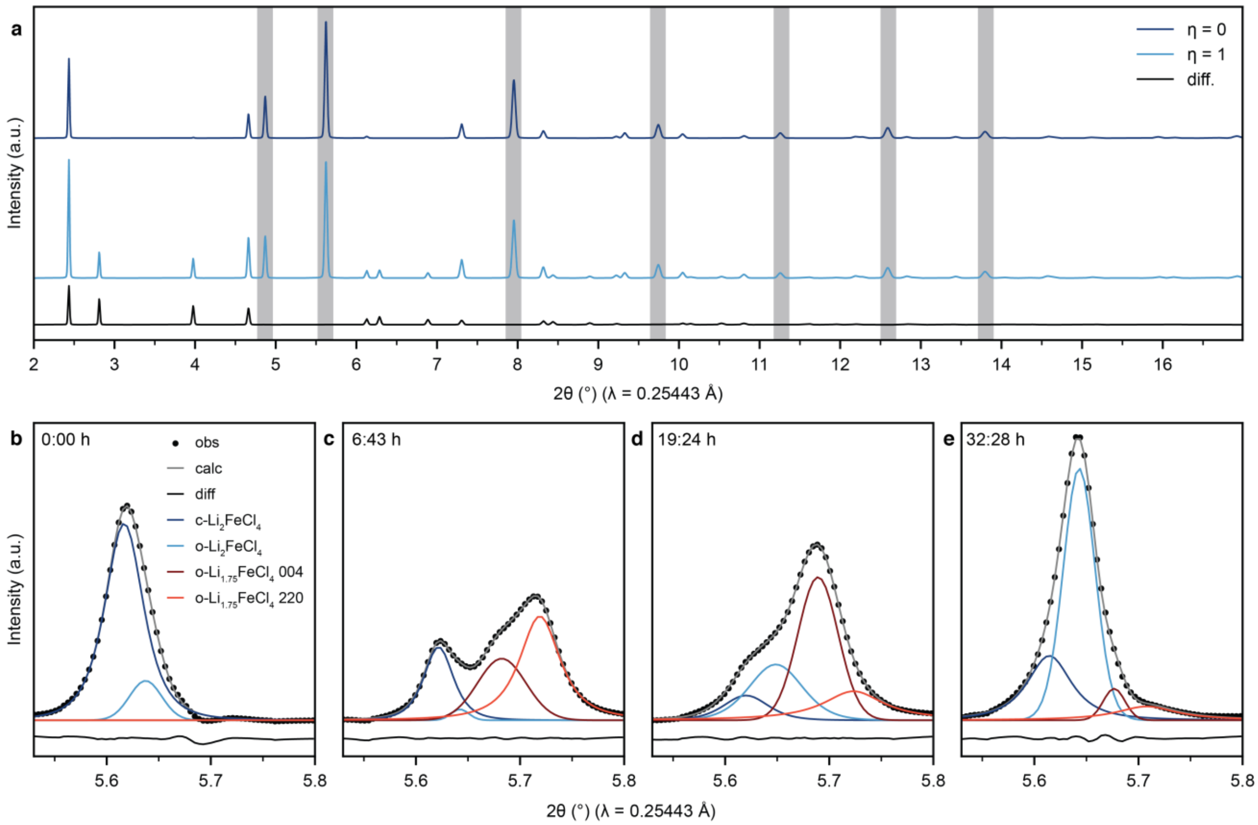

Figure S13. (a) Simulated XRD pattern of fully ordered Li_2FeCl_4 (η = 1) and fully disordered Li_2FeCl_4 (η = 0), as well as their difference, with the reflections that are unaffected by ordering highlighted in grey. (**b-e**) Peak fit of the 220/004 reflection (expressed in the *Imma* space group) at selected points during electrochemical cycling.

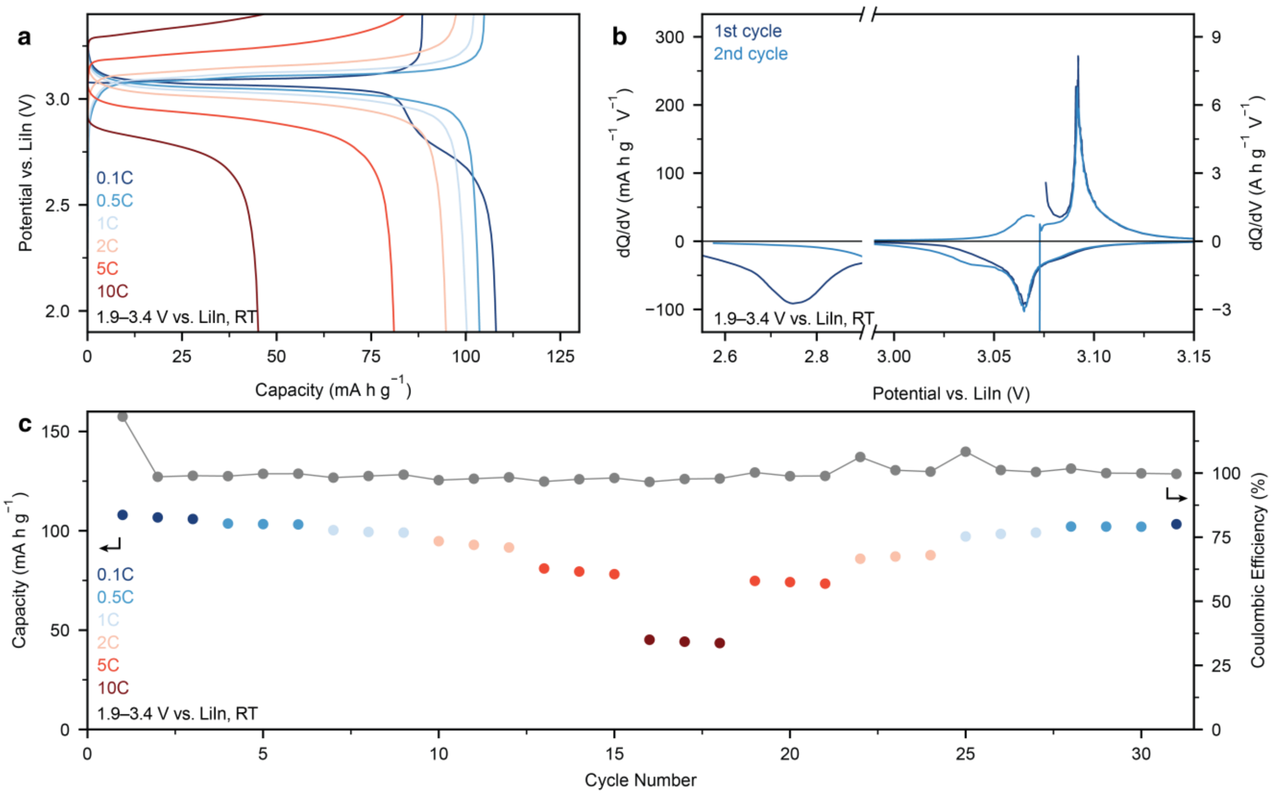

Figure S14. Voltage profile (**a**), dQ/dV plot (**b**) and cycling stability (**c**) of all-solid-state batteries with cathodes containing $Li_{1.75}FeCl_4$ at different rates.

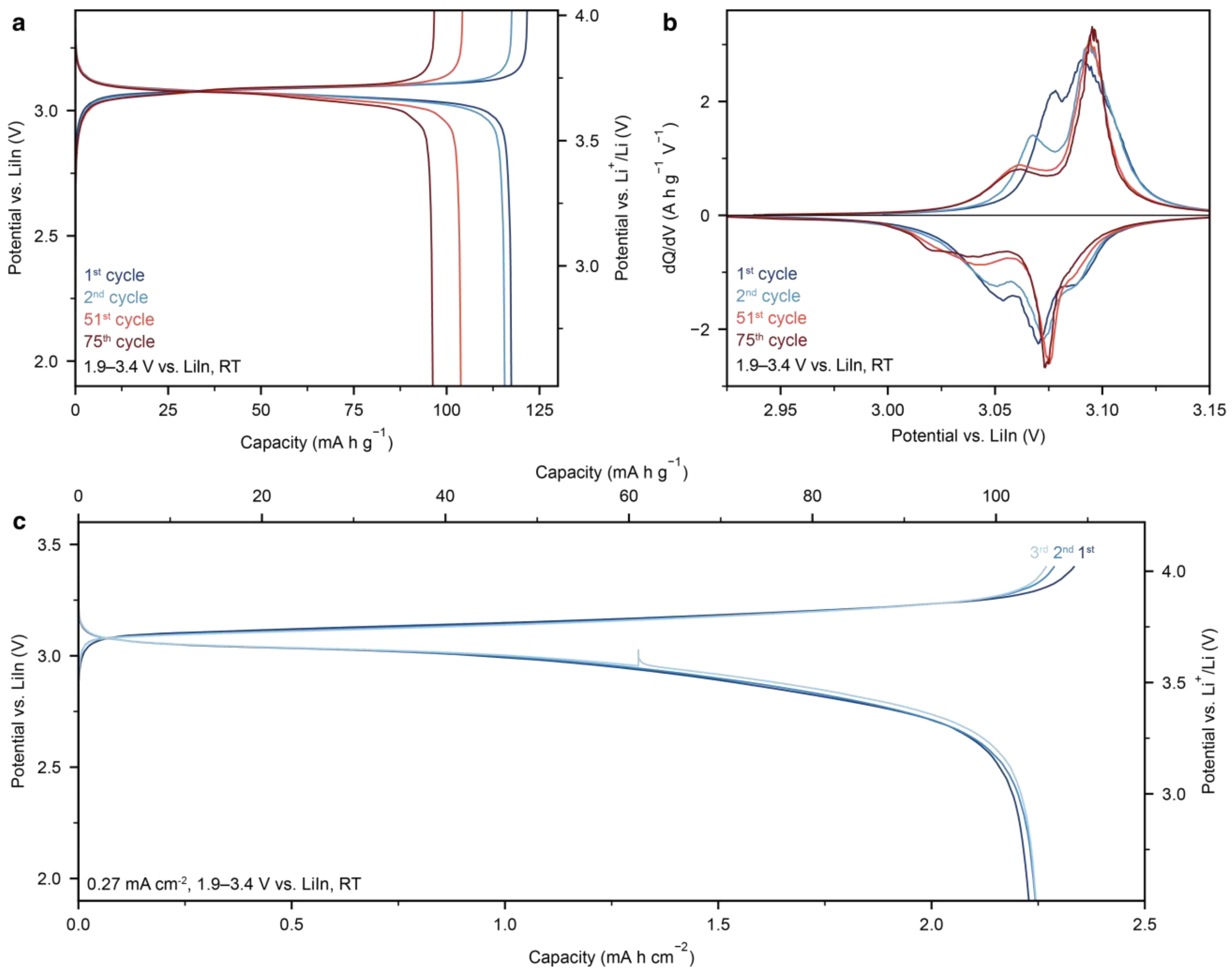

Figure S15. Voltage profile (**a**) and dQ/dV plots (**b**) for selected cycles of the all-solid-state batteries with cathodes containing Li_2FeCl_4 shown in Figure 5B, C. (**c**) Voltage profile for the 3 precycles of the all-solid-state battery with a cathode containing Li_2FeCl_4 displayed in Figure 5D.

REFERENCES

(1) Tanibata, N.; Kato, M.; Takimoto, S.; Takeda, H.; Nakayama, M.; Sumi, H. High Formability and Fast Lithium Diffusivity in Metastable Spinel Chloride for Rechargeable All-Solid-State Lithium-Ion Batteries. *Adv. Energy Sust. Res.* **2020**, *1* (1), 2000025. DOI: 10.1002/aesr.202000025.

(2) Schlem, R.; Muy, S.; Prinz, N.; Banik, A.; Shao-Horn, Y.; Zobel, M.; Zeier, W. G. Mechanochemical Synthesis: A Tool to Tune Cation Site Disorder and Ionic Transport Properties of Li_3MCl_6 (M = Y, Er) Superionic Conductors. *Adv. Energy Mater.* **2020**, *10* (6), 1903719. DOI: 10.1002/aenm.201903719.

(3) Hammer, R. R.; Gregory, N. W. An Effusion Study of the Simultaneous Vaporization and Decomposition of Solid Iron(III) Chloride. *The Journal of Physical Chemistry* **1962**, *66* (9), 1705-1707. DOI: 10.1021/j100815a035.

(4) Dyadkin, V.; Pattison, P.; Dmitriev, V.; Chernyshov, D. A new multipurpose diffractometer PILATUS@SNBL. *J. Synchrotron Rad.* **2016**, *23* (3), 825-829. DOI: 10.1107/S1600577516002411.

(5) Toby, B. H.; Von Dreele, R. B. GSAS-II: the genesis of a modern open-source all purpose crystallography software package. *J. Appl. Crystallogr.* **2013**, *46* (2), 544-549. DOI: 10.1107/S0021889813003531.

(6) Schindelin, J.; Arganda-Carreras, I.; Frise, E.; Kaynig, V.; Longair, M.; Pietzsch, T.; Preibisch, S.; Rueden, C.; Saalfeld, S.; Schmid, B.; et al. Fiji: an open-source platform for biological-image analysis. *Nat. Methods* **2012**, *9* (7), 676-682. DOI: 10.1038/nmeth.2019.

(7) Fehrenbach, J.; Weiss, P.; Lorenzo, C. Variational Algorithms to Remove Stationary Noise: Applications to Microscopy Imaging. *IEEE Trans. Image Process.* **2012**, *21* (10), 4420-4430. DOI: 10.1109/TIP.2012.2206037.

(8) Jamnik, J.; Maier, J. Treatment of the Impedance of Mixed Conductors Equivalent Circuit Model and Explicit Approximate Solutions. *J. Electrochem. Soc.* **1999**, *146* (11), 4183. DOI: 10.1149/1.1392611.

(9) Jamnik, J.; Maier, J. Generalised equivalent circuits for mass and charge transport: chemical capacitance and its implications. *Phys. Chem. Chem. Phys.* **2001**, *3* (9), 1668-1678, 10.1039/B100180I. DOI: 10.1039/B100180I.

(10) Lai, W.; Haile, S. M. Impedance Spectroscopy as a Tool for Chemical and Electrochemical Analysis of Mixed Conductors: A Case Study of Ceria. *J. Am. Ceram. Soc.* **2005**, *88* (11), 2979-2997. DOI: 10.1111/j.1551-2916.2005.00740.x.

(11) Boukamp, B. A. A Nonlinear Least Squares Fit procedure for analysis of immittance data of electrochemical systems. *Solid State Ion.* **1986**, *20* (1), 31-44. DOI: 10.1016/0167-2738(86)90031-7.

(12) Macdonald, J. R.; Potter, L. D. A flexible procedure for analyzing impedance spectroscopy results: Description and illustrations. *Solid State Ion.* **1987**, *24* (1), 61-79. DOI: 10.1016/0167-2738(87)90068-3.

(13) Kanno, R.; Takeda, Y.; Takada, K.; Yamamoto, O. Phase diagram and ionic conductivity of the lithium chloride-iron(II) chloride system. *Solid State Ion.* **1983**, *9-10*, 153-156. DOI: 10.1016/0167-2738(83)90225-4.

(14) Lutz, H. D.; Kuske, P.; Wussow, K. Neue Lithiumchlorid-Suzukiphasen: Li_6MCl_8 (M = Fe, Co, Ni). *Z. Anorg. Allg. Chem.* **1987**, *553* (10), 172-178. DOI: 10.1002/zaac.19875531020.

(15) Landau, L. D.; Lifshitz, E. M. *Statistical Physics: Volume 5*; Pergamon, 1980.

(16) Adamič, M.; Talian, S. D.; Sinigoj, A. R.; Humar, I.; Moškon, J.; Gaberšček, M. A Transmission Line Model of Electrochemical Cell's Impedance: Case Study on a Li-S System. *J. Electrochem. Soc.* **2019**, *166* (3), A5045. DOI: 10.1149/2.0061903jes.

(17) Bumberger, A. E.; Steinbach, C.; Ring, J.; Fleig, J. Mass and Charge Transport in $Li_{1-\delta}CoO_2$ Thin Films─A Complete Set of Properties and Its Defect Chemical Interpretation. *Chem. Mater.* **2022**, *34* (23), 10548-10560. DOI: 10.1021/acs.chemmater.2c02614.

(18) Bumberger, A. E.; Nenning, A.; Fleig, J. Transmission line revisited – the impedance of mixed ionic and electronic conductors. *Phys. Chem. Chem. Phys.* **2024**, *26* (21), 15068-15089, 10.1039/D4CP00975D. DOI: 10.1039/D4CP00975D.

(19) Huggins, R. A. Simple method to determine electronic and ionic components of the conductivity in mixed conductors a review. *Ionics* **2002**, *8* (3), 300-313. DOI: 10.1007/BF02376083.

(20) Kraft, M. A.; Ohno, S.; Zinkevich, T.; Koerver, R.; Culver, S. P.; Fuchs, T.; Senyshyn, A.; Indris, S.; Morgan, B. J.; Zeier, W. G. Inducing High Ionic Conductivity in the Lithium Superionic Argyrodites $Li_{6+x}P_{1-x}Ge_xS_5I$ for All-Solid-State Batteries. *J. Am. Chem. Soc.* **2018**, *140* (47), 16330-16339. DOI: 10.1021/jacs.8b10282.

(21) Huang, Z.; Yoshida, S.; Akamatsu, H.; Hayashi, K.; Ohno, S. $NaMCl_6$ (M = Nb and Ta): A New Class of Sodium-Conducting Halide-Based Solid Electrolytes. *ACS Mater. Lett.* **2024**, *6* (5), 1732-1738. DOI: 10.1021/acsmaterialslett.4c00315.

(22) Siebenhofer, M.; Baiutti, F.; de Dios Sirvent, J.; Huber, T. M.; Viernstein, A.; Smetaczek, S.; Herzig, C.; Liedke, M. O.; Butterling, M.; Wagner, A.; et al. Exploring point defects and trap states

in undoped $SrTiO_3$ single crystals. *J. Eur. Ceram. Soc.* **2022**, *42* (4), 1510-1521. DOI: 10.1016/j.jeurceramsoc.2021.10.010.

(23) Liu, Z.; Zhang, G.; Pepas, J.; Ma, Y.; Chen, H. Li_2FeCl_4 as a Cost-Effective and Durable Cathode for Solid-State Li-Ion Batteries. *ACS Energy Lett.* **2024**, *9* (11), 5464-5470. DOI: 10.1021/acsenergylett.4c02376.

(24) Peng, D.; Li, R.; Xu, K.; Si, R.; Zhang, Z.; Hu, Y.-S. A Low-Strain Lithium Cathode Material $Li_{2-2x}Fe_{1+x}Cl_4$ for Halide-Based All-Solid-State Batteries. *ACS Energy Lett.* **2025**, 1421-1429. DOI: 10.1021/acsenergylett.4c03147.

(25) Eckhardt, J. K.; Klar, P. J.; Janek, J.; Heiliger, C. Interplay of Dynamic Constriction and Interface Morphology between Reversible Metal Anode and Solid Electrolyte in Solid State Batteries. *ACS Appl. Mater. Interfaces* **2022**, *14* (31), 35545-35554. DOI: 10.1021/acsami.2c07077.

(26) Asano, T.; Sakai, A.; Ouchi, S.; Sakaida, M.; Miyazaki, A.; Hasegawa, S. Solid Halide Electrolytes with High Lithium-Ion Conductivity for Application in 4 V Class Bulk-Type All-Solid-State Batteries. *Adv. Mater.* **2018**, *30* (44), 1803075. DOI: 10.1002/adma.201803075.

(27) Okur, F.; Sheima, Y.; Zimmerli, C.; Zhang, H.; Helbling, P.; Fäh, A.; Mihail, I.; Tschudin, J.; Opris, D. M.; Kovalenko, M. V.; et al. Nitrile-functionalized Poly(siloxane) as Electrolytes for High-Energy-Density Solid-State Li Batteries. *ChemSusChem* **2024**, *17* (3), e202301285. DOI: 10.1002/cssc.202301285.

(28) Lutz, H. D.; Pfitzner, A.; Cockcroft, J. K. Structural Phase Transition and Nonstoichiometry of Li_2FeCl_4—Neutron Diffraction Studies. *J. Solid State Chem.* **1993**, *107* (1), 245-249. DOI: 10.1006/jssc.1993.1344.

(29) Ott, H. Die Raumgitter der Lithiumhalogenide. *Phys. Z.* **1923**, *24*, 209-213.

(30) van Loon, C. J. J.; de Jong, J. Some chlorides with the inverse spinel structure. *Acta Crystallogr. B* **1975**, *31* (10), 2549-2550. DOI: 10.1107/S0567740875008114.

(31) Palvadeau, P.; Venien, J. P.; Spiesser, M.; Rouxel, J. Characterization of $LiFeCl_4$ and $AgFeCl_4$ ionic conductors. *Solid State Ion.* **1982**, *6* (3), 231-236. DOI: 10.1016/0167-2738(82)90044-3.
